# Present-day Earth mantle structure set up by crustal pollution of the basal magma ocean

**DOI:** 10.1126/sciadv.adu2072

**Published:** 2025-07-18

**Authors:** Maxim D. Ballmer, Rob J. Spaargaren, Ananya Mallik, Antonio Manjón-Cabeza Córdoba, Miki Nakajima, Kenny Vilella

**Affiliations:** ^1^ Dept. Earth Sciences, University College London, London WC1E 6BT, UK.; ^2^ Dept. Earth and Planetary Sciences, ETH Zürich, Zürich 8092, Switzerland.; ^3^ Kapteyn Astronomical Institute, Univ. Groningen, Groningen 9700AV, Netherlands.; ^4^ Dept. Geosciences, University of Arizona, 1040 E. 4th street, Tucson, AZ 85721, USA.; ^5^ Dept. Earth and Environmental Sciences, University of Rochester, Rochester, NY 14627, USA.; ^6^ JSPS International Research Fellow, Hokkaido University, Sapporo, Japan.

## Abstract

The crystallization of a global magma ocean during early terrestrial planet evolution and the subsequent segregation of a longer-lived “basal magma ocean” (BMO) atop the core set up the evolution of the mantle-atmosphere system. Although seismic evidence for a BMO exists on present-day Mars and the Moon, the Earth’s BMO is (near-)completely solidified. Seismically observed “large low-velocity provinces” (LLVPs) are thought to have resulted from the canonical “fractional” style of BMO crystallization. However, we show using thermodynamic modeling that BMO fractional crystallization yields lowermost-mantle densities much higher than those of LLVPs. In turn, pollution of the BMO by progressive addition of recycled basaltic crust and related “reactive crystallization” can reconcile LLVP volumes, densities, and compositions. This model also makes testable predictions of the compositions of “ultralow-velocity zones,” enigmatic deep Earth seismic domains, and possible BMO remnants. The critical role of crustal pollution elucidates the survival of a BMO on Mars, but implies an Earth-like fate for any Venusian BMO.

## Teaser

Basal magma ocean mixing with subducted crust explains solidification of Earth BMO and seismic anomalies in the lowermost mantle.

## INTRODUCTION

The formation of a basal magma ocean (BMO) in the early Earth is a natural consequence of planetary differentiation. The accretion and segregation of the planet releases massive extents of potential energy, melting most if not all of the mantle as a global magma ocean. For dominant crystal settling (or “fractional crystallization”) of the global magma ocean (*1*), a BMO with a thickness of ~350 km segregates from the rest of the mantle because magmas at lowermost-mantle conditions are denser than corresponding crystals (*2*–*4*). A BMO with such an origin would initially assume bulk-silicate Earth (i.e., similar to present-day upper-mantle “pyrolitic” compositions) (*5*) compositions (*2*, *3*) but may also preserve proto-Earth signatures (*6*, *7*). For dominant “batch crystallization” of the global magma ocean due to ultrafast turbulent cooling (*8*), a lower-mantle crystal mush is formed and eventually spawns a thick (~900 km) Fe-enriched BMO (*3*). Alternatively, the overturn and deep re-melting of early crust can generate (or contribute to) a silica-enriched, or roughly spoken “basaltic”, BMO (*9*, *10*). In either case, a BMO with variable initial size and composition is virtually inevitably formed, eventually cooling over billions of years (*2*). Related convection within such an early-Earth BMO may help to explain paleomagnetic evidence for an early geodynamo (*11*, *12*).

Seismic data provide evidence for the preservation of a highly Fe-enriched BMO on present-day Mars (*13*–*15*) and a similar partially molten basal layer on the Moon (*16*, *17*), further supporting the BMO hypothesis. However, although BMO formation tends to be more likely for larger planets (*2*), an analogous (partially) liquid rocky layer covering the Earth’s core-mantle boundary (CMB) can be ruled out geophysically (*18*). To resolve this discrepancy, we here compute the crystallization sequence of the BMO using a thermodynamic model (*19*) (see Materials and Methods) and explore the related implications for deep Earth structure.

## RESULTS AND DISCUSSION

### BMO fractional crystallization

**Fig. 1.**
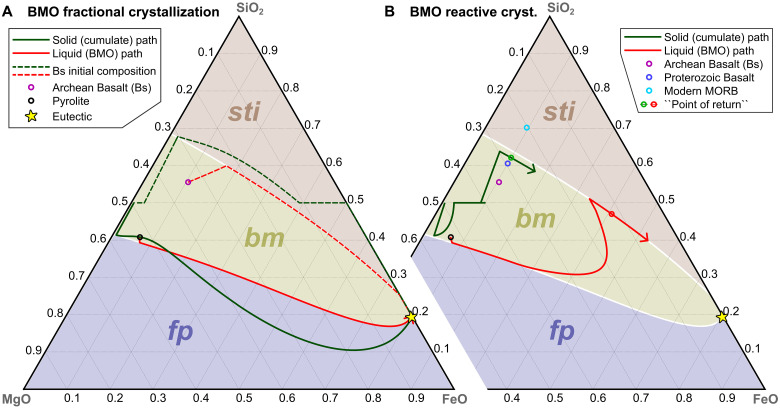
Crystallization sequences for fractional and reactive crystallization of the BMO. Ternary phase diagrams [from ref. (*19*) at 130 GPa] with mineral stability fields (colors) as labeled. Lines show the progressive (i.e., from left to right) compositional evolution of the liquid BMO and corresponding solid crystal cumulates. White lines mark cotectic valleys. Fractional crystallization (**A**) ultimately yields extremely iron-enriched, (near-)eutectic liquid and solid compositions. Reactive crystallization (**B**) yields low-to-moderately (arrows) iron-enriched liquid and solid compositions (because BMO/cumulate compositional evolution terminates at the red/green arrows).

**Fig. 2.**
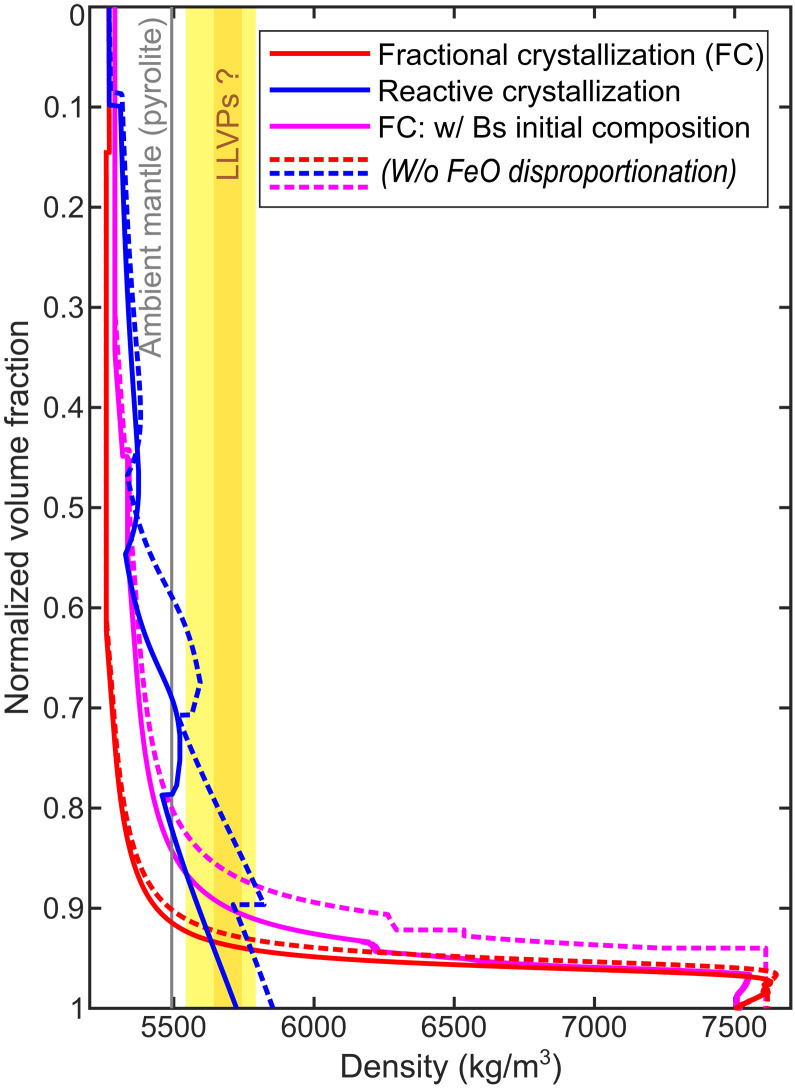
Density profiles for fractional and reactive crystallization of the BMO. Cumulate density profiles for BMO fractional crystallization reach up to extremely high values (~7500 kg/m^3^), independent of BMO initial composition (red/pink lines). Density profiles for BMO reactive crystallization instead reach cumulate densities of only 5700~5800 kg/m^3^ (blue lines). The effects of FeO disproportionation (solid versus dashed lines) are second order. Volume fractions (*y* axis) are normalized by the total cumulate volume for each case. For comparison, estimates for LLVP intrinsic density anomalies from various geodynamic modeling studies are shown as yellow bar [see Supplementary Discussion; orange bar from ref. (*22*)]. For substantially higher density anomalies, a global layer instead of LLVP-like piles should be formed (*20*, *21*).

For the BMO, upward crystal setting is promoted by slow cooling, clearly favoring fractional (over batch) crystallization (*1*, *2*). During BMO fractional crystallization, the first mineral phase to crystallize is bridgmanite (bm) (*3*, *19*). As bm is extracted fractionally, a BMO with pyrolytic initial composition (evolves such that eventually bm+ferropericlase (fp) crystallize over most of BMO evolution (Fig. 1A, solid lines). For basaltic initial compositions, bm+stishovite (sti) crystallize over most of BMO evolution (Fig. 1A, dashed lines). In both cases, the BMO becomes progressively Fe-enriched during crystallization (Fig. 1A, red solid/dashed lines), culminating at highly Fe-enriched “eutectic” compositions (Fig. 1A, yellow star). The eutectic is the composition with the lowest melting temperatures, and is inevitably reached during progressive fractional crystallization. Accordingly, final-stage crystal cumulates also assume highly Fe-enriched eutectic compositions (Fig. 1A, green lines). About ~10% of crystallized cumulates achieve near-eutectic compositions with densities >1000 to ~2140 kg/m^3^ higher than the ambient mantle (Fig. 2, red/pink lines).

**Fig. 3.**
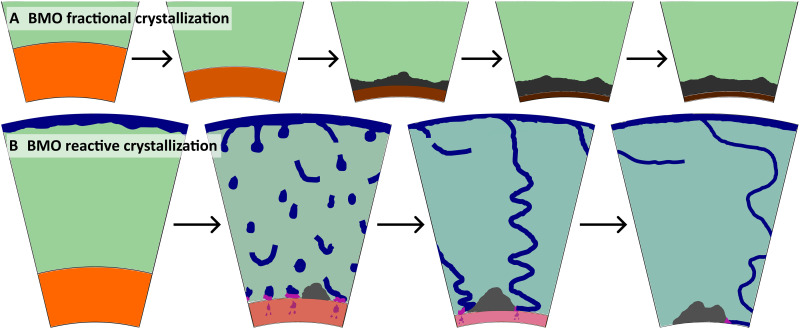
Conceptual visualization of the consequences of BMO crystallization for Earth evolution and compositional structure. (**A**) BMO fractional crystallization. (**B**) BMO reactive crystallization. The BMO (orange-to-brown) becomes progressively enriched in iron in (A) or polluted by molten basaltic crust (pink) in (B), forming variably dense cumulates upon crystallization (shades of gray). Because of crustal pollution of the BMO (B), the ambient mantle (green) becomes oxidized (teal) over time. The thickness of basaltic crust (blue) is not to scale.

Because of these predicted extreme density anomalies, the consequences of BMO fractional crystallization are at odds with Earth structure. Such a dense and highly iron-enriched layer (Figs. 1B and 2) cannot be entrained and stirred into the mantle by subsequent solid-state convection (*20*–*22*). It would remain gravitationally stable in the lowermost mantle, stabilizing a global dense layer with a volume of ~10% of the initial BMO (Fig. 2, blue lines) until the present day (*20*, *22*–*24*), inconsistent with seismic constraints. The seismic properties of such a solidified layer with a composition similar to wüstite (FeO; Fig. 1A) would be similar to those of the observed “ultralow-velocity zones” (ULVZs) (*25*), but the ULVZs are much too small (*18*, *26*) to accommodate ~10% of the initial BMO (*3*). Although there is evidence of large seismic anomalies in the lowermost mantle [e.g., large low-velocity provinces (LLVPs)], which are interpreted as thermochemical piles and have been conceptually related to BMO crystallization (*2*), their estimated intrinsic density excess is much smaller (<300 kg/m^3^; orange/yellow bar in Fig. 2) than that predicted for fractional crystallization (*20*–*22*, *27*–*31*). Moreover, the eutectic temperature of the crystallizing BMO (*19*, *32*, *33*) is notably lower than most estimates for present-day CMB temperatures (*34*–*36*), particularly if enrichment of minor elements and volatiles is taken into account (*2*, *37*). Thus, a BMO with up to ~10% of its initial volume should actually still be present at the CMB in the fractional-crystallization scenario (Fig. 3A). However, neither a (partially) liquid nor a solid global basal layer (i.e., more than 1 to 2 km thick) exists on our planet, ruled out by seismic observations (*18*).

An additional process that can remove iron from the BMO and therefore promote solidification is FeO disproportionation (*38*). As the BMO crystallizes, bm cumulates incorporate Al as Al^3+^Fe^3+^O_3_, for which any available Fe^2+^O is oxidized (*38*, *39*). To maintain charge balance, another Fe atom must be reduced. We account for the effects of this process by removing one elemental Fe^0^ to the core for any two Al atoms incorporated into bm (see Materials and Methods). We find that this iron removal reduces the thickness of the highly enriched layer, but notably not its density anomaly (Fig. 2, red/pink dashed lines). Accordingly, this process is insufficient to reconcile BMO fractional crystallization with present-day Earth’s structure; alternative scenarios need to be explored.

### BMO reactive crystallization

Here, we propose a “reactive-crystallization” mechanism that is driven by the continuous recycling of basaltic crust and chemical interaction of this crust with the BMO. In the modern Earth, oceanic crust is efficiently cycled into the mantle during subduction and tends to segregate from the ambient mantle to settle at the CMB (*24*, *40*, *41*). Although the tectonic regime(s) of the early Earth remain(s) debated, the proposed regimes involve efficient crustal recycling (*42*–*46*), consistent with geochemical constraints (*47*). As oceanic crust has likely been three to four times thicker in the early Earth (*48*, *49*), crustal recycling may have been even more efficient than in the present day (*50*). Moreover, early CMB temperatures have been higher (*51*) and thus above the solidus of basaltic crustal rocks (*52*), promoting melting at the BMO-mantle boundary. Any such deep mantle melts would have mixed or at least chemically equilibrated (“reacted”) with the BMO (Fig. 3B). We quantify the consequences of such crustal addition (or “crustal pollution”) and BMO reactive crystallization, assuming that crustal material (with time-evolving composition; see Fig. 1B and Materials and Methods) is continuously mixed into the BMO as it cools. In this case, the crystallizing BMO is an open system, as opposed to being a closed system for fractional crystallization.

**Fig. 4.**
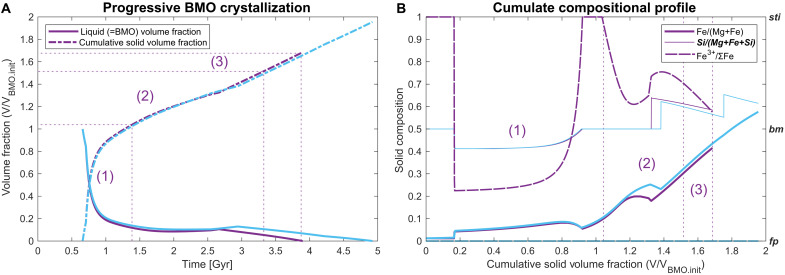
Progression of BMO crystallization over time and cumulate compositional profiles. Progression of crystallization (**A**) and cumulate compositional profiles (**B**) are shown for the reference case (purple) and a corresponding case without FeO disproportionation (cyan). The BMO (liquid) compositional evolution during crystallization and the density profile of the reference case are shown in fig. S2B and Fig. 5 (purple lines), respectively (also see Fig. 1B and Fig. 2). For the reference case, (1) the positively (<−50 kg/m^3^), (2) near-neutral (gray band in Fig. 5), and (3) negatively (>50 kg/m^3^) buoyant parts of the BMO cumulate sequence are labeled (numbers).

We find that, in contrast to fractional crystallization, the BMO does not reach extremely enriched eutectic compositions in this reactive-crystallization scenario. Figure 1B shows that a BMO with initially pyrolytic composition (black circle) evolves similarly as for fractional crystallization at first (red line) due to the dominant effects of cooling. First, bm and then bm+fp are crystallized. However, as cooling slows down over time (fig. S1) and crustal material is continuously added, the BMO becomes progressively enriched in silica, leaving the bm+fp phase boundary. From this point, exclusively bm crystallizes, but the BMO continues to become further enriched in silica (intermittently actually growing in volume; purple solid line in Fig. 4A) until it eventually crystallizes bm+sti. Upon further cooling, the BMO shrinks again, travelling down the bm+sti cotectic line (Fig. 1B), where both these minerals crystallize together (here in an ~3:1 ratio; Fig. 4B). Last, the BMO crosses the “point of return” (red circle in Fig. 1B). Beyond this point, the cumulates, which crystallize upon crustal pollution, become more iron enriched than the continuously added crustal material (brown circle in Fig. 1B). Thus, iron is effectively pumped out of the BMO into these cumulates and eventually into the mantle. At this point, the BMO would completely crystallize chemically due to crustal pollution and related reaction even if no further cooling occurred (i.e., if BMO temperatures were fixed at ~4150 K). In our model, however, cooling continues (fig. S1), such that the BMO evolves further than the “point of return.” Nevertheless, the BMO fully solidifies well before reaching eutectic compositions (Fig. 1B), such that maximum cumulate density anomalies do not exceed 200~300 kg/m^3^ (i.e., an order of magnitude lower than for fractional crystallization), consistent with estimates for LLVP intrinsic density anomalies (Fig. 2).

FeO disproportionation further helps to remove iron from the crystallizing BMO into the core. Accounting for this process reduces the lifetime of the BMO, as well as the total volume, maximum Fe enrichment, and maximum density anomaly of the cumulates (Fig. 4). FeO disproportionation is more efficient for reactive crystallization than for fractional crystallization because the continuously added basaltic material is enriched in Al (table S3), driving disproportionation (*38*, *53*). Nevertheless, most BMO iron (i.e., >80% of initial plus crustal-pollution budget) is still pumped into the mantle as oxidized cumulates (Fe^3+^/ΣFe ≈ 0.6; Fig. 4B). Accordingly, with or without considering FeO disproportionation, maximum density anomalies in the crystal cumulate pile in the lowermost mantle do not exceed ~350 kg/m^3^ (Fig. 2).

**Fig. 5.**
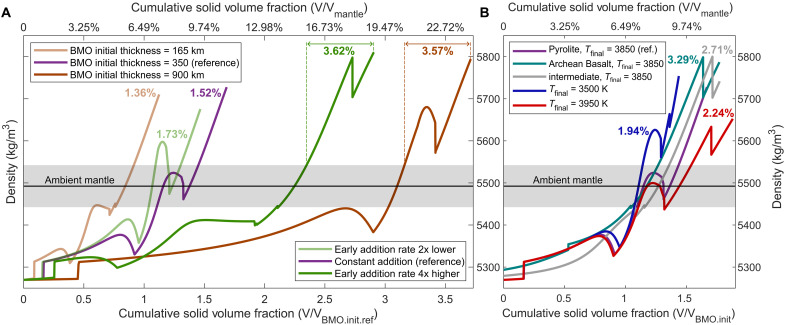
Parameter sensitivity of BMO cumulate density profiles. Cumulate densities are shown as a function of cumulative solid fraction for different (**A**) BMO initial thicknesses and crustal addition rates and (**B**) for various BMO initial compositions (table S3) and cooling histories (fig. S1). Cumulative solid fraction relates to time as shown in Fig. 4A and figs. S2A to S5A (time generally increases to the right for any given case). The reference case (purple) is the same as the case with FeO disproportionation in Figs. 1, 2, and 4. Numbers indicate the mantle volume fraction of intrinsically dense (>50 kg/m^3^) cumulates at the present day [dashed lines in (A) indicate how these volumes are derived]. The gray bar indicates a density range around ambient mantle pyrolite. Brown lines in (A) can also be understood as cases with different Φ at a fixed initial thickness of 350 km (table S1). Light and dark green lines represent cases with early addition rates (<2.5 Gyr) of 0.5Φ and 4Φ, respectively.

Such moderate maximum density anomalies occur for all our reactive-crystallization cases. In our reference case (Figs. 4 and 5, purple line), we consider a BMO initially ~350 km thick and with pyrolytic composition (i.e., ~bulk silicate Earth) (*2*, *3*). Crustal addition rates, Φ, are fixed over time at present-day subduction fluxes (table S2), but we explore cases with variable early [<2.5 billion years ago (Ga)] crustal addition rates, Φ*_early_*. BMO cooling histories are anchored by the present-day CMB temperature, *T_final_* = 3850 K (fig. S1). For a detailed discussion of the parameter sensitivity of our models, see Supplementary Results. In summary, we find that the total volume of cumulates (colored numbers in Fig. 5) increases with the (early) crustal addition rate and lifetime of the BMO. In turn, BMO lifetimes decrease with early addition rates (fig. S2), and increase with *T_final_* and BMO initial size and Fe content (figs. S3 to S5). Average cumulate oxidation due to FeO disproportionation also varies between cases shown in Fig. 5 and figs. S2 to S6 (0.42 < Fe^3+^/ΣFe < 0.89). However, we emphasize that cumulate mineralogy, Fe contents, and density anomalies are largely independent of crustal-addition or BMO-cooling history (Fig. 5 and figs. S2 to S4), as well as BMO initial thickness (Fig. 5A and fig. S4) or composition (Fig. 5B and fig. S5). For all our cases, the maximum predicted density anomalies are moderate (reaching just 200~300 kg/m^3^), consistent with the structure of Earth’s lower mantle:

### Consequences for earth evolution and structure

The BMO cumulate sequence can be separated into three parts (Fig. 4). The first part is depleted (e.g., in iron) and notably less dense than the ambient mantle. It is expected to actively rise to the upper mantle and/or being stirred into the mantle. The second part of the cumulate sequence is enriched in silica (dominantly bm and ≤25% sti) with near-neutral (gray band in Fig. 5) density anomalies. This material would float in but only partly mixed into the mantle within a few billion years, due to its high intrinsic viscosity (*54*).

The first, buoyant part of BMO cumulates (numbers in Fig. 4) may contribute to the stabilization of continental/cratonic keels in the Hadean/Archean (*55*). Entrained BMO cumulates (i.e., with high Fe^3+^/ΣFe) may moreover contribute to the progressive oxidation of the (upper) mantle over time (*56*–*58*). Accordingly, the deep Earth oxygen cycle may be powered by BMO crustal pollution, potentially linked to the Great Oxidation Event of the atmosphere with implications for the evolution of higher life (*56*–*59*). In turn, the nonentrained portion of the second part of the cumulate sequence is a good candidate material to be preserved as mid-sized to large bridgmanitic blobs (*54*, *60*, *61*), also referred to as “BEAMS” (*54*, *61*). These high-viscosity bridgmanitic domains (*62*, *63*) can account for widespread seismic reflectors (*64*), slab stagnation (*65*) and deflection of plume upwellings (*66*) in the mid-mantle. The predicted abundance of stishovite in such blobs (Fig. 4B) can further promote floating and hence preservation of blobs in the mid-mantle owing to a density crossover relative to the ambient mantle at ~1600 km depth (*67*).

Last, the third part of the cumulate sequence is also enriched in bm but notably exhibits progressive iron enrichments (Fig. 4). Density anomalies of this material are therefore moderately positive, ranging from 50 to 200 kg/m^3^, up to maximally ~300 kg/m^3^ in some cases, but usually 100~150 kg/m^3^ on average (Fig. 5 and figs. S2C to S6C). Such moderately dense materials will not form a stable global layer that covers most of the CMB but rather long-lived isolated thermochemical piles (yellow/orange bar in Fig. 2 and Supplementary Discussion), as shown by many previous geodynamic studies (*20*, *22*, *23*, *68*, *69*). Therefore, the third part of the BMO reactive crystallization cumulate sequence is a good candidate to account for seismically observed LLVPs (*23*, *27*, *29*).

The predicted volumes and compositions of the third part of BMO cumulates are consistent with LLVP seismic signatures: The distinct response of LLVPs to traversing shear versus pressure waves points to bm enrichment, moderate iron enrichment, and high oxidation (*27*, *28*), as predicted here (Fig. 4B and figs. S2D to S6D). Our predicted volumes of intrinsically dense cumulates mostly range between 1.5 and 3.5% of that of the mantle (numbers in Fig. 5) (or up to 4.8% depending on bm-melt iron partitioning; fig. S6), consistent with seismic estimates for LLVP volumes. Seismic estimates range from 2~3% (*70*) up to ~8% (*71*), but at least the latter estimate likely includes a purely thermal region around chemically distinct piles (*29*, *69*, *72*).

As proposed in ref. (*2*), the Earth’s BMO may not be completely crystallized at the present day. A very small partially molten remnant may be seismically visible as ULVZs (*2*, *73*). For any BMO crystallization scenario, volatiles and incompatible elements would be concentrated in the last small remnant, preventing complete solidification. If this hypothesis is correct, BMO reactive crystallization makes geophysically testable predictions of ULVZ major-element compositions. The predicted ULVZ compositions are highly enriched in Fe with near-pyrolitic SiO_2_ (Fe# ≈ 90; see tip of red arrow in Fig. 1B), consistent with mineral-physics estimates (*74*), although uncertainties persist (*26*).

Crustal pollution of the BMO during reactive crystallization has further important geochemical implications. Early depleted cumulates from an initially enriched BMO (e.g., in the overturn scenario of BMO formation) can account for the coupled Hf-W-Nd isotope systematics of Archean igneous rocks (*75*). Furthermore, the rather sharp decline of isotopic anomalies with primordial origin (e.g., ^142^Nd/^144^Nd, ^182^W/^184^W) in the late Archean (2.0~2.5 Ga) (*76*, *77*) overlaps with the switch from buoyant to intrinsically dense cumulates in our reference case (Fig. 4A). Thus, the decline of these anomalies may be primarily related to the timing of BMO solidification and only secondarily to progressive mantle mixing (*77*). Improving geochemical data may help to constrain BMO formation scenarios and core-mantle interaction (*78*). Moreover, the pollution of the BMO by recycled crust may reconcile the proposed primordial BMO origin of LLVPs (*2*) with the hot spot isotopic record. Hot spot–feeding mantle plumes grazing LLVP sample mantle reservoir(s) with primitive noble-gas signatures (*37*, *79*) but are dominated by recycled isotopic and trace-element fingerprints (*80*).

FeO disproportionation as driven by BMO crustal pollution also adds pure iron at the top of the core. The related total mass of added iron corresponds to ~14% of Earth’s inner core mass for our reference case, or ~41% for large BMO thicknesses (900 km) combined with high Φ*_early_* (4Φ). Accordingly, reactive crystallization can release up to ~41% of the potential (or convective) energy of inner-core growth that is available to drive the geodynamo. Thus, (early) crustal recycling can substantially contribute to the (early) Earth’s magnetic field, perhaps along with other mechanisms (*11*, *81*).

### Implications for terrestrial planets

Here, we have shown that fractional crystallization of the BMO leads to a highly Fe-enriched layer that covers the CMB, inconsistent with geophysical constraints. As the formation of a BMO early in the history of an Earth-sized planet is almost inevitable (*82*) and further supported by seismic evidence for a BMO on present-day Mars (*13*–*15*), an additional mechanism is required. We demonstrate that BMO reactive crystallization driven by crustal pollution, as is expected to occur on a tectonically active planet, can reconcile present-day Earth structure.

Although estimated BMO cooling timescales span billions of years (*2*), BMO crystallization and reaction with recycled crust may, in principle, have also occurred consecutively, instead of simultaneously as suggested here. However, such a scenario requires very fast early core cooling and, subsequently, efficient solid-solid reaction/metasomatism in a relatively cool lowermost mantle. In turn, fluxing massive amounts of (partially) molten crust through an already evolved BMO can explain how such a BMO can efficiently crystallize (chemically) in the first place, instead of being stabilized (thermally) by ever-increasing concentrations of heat-producing elements.

BMO reactive crystallization due to crustal pollution can further provide a unified model of Earth’s present-day lower-mantle structure, naturally explaining the coupled origins of moderately enriched LLVPs and (partially) molten ULVZs. It can explain why Earth’s present-day CMB is not covered by a preserved BMO, or a (thick) very dense solid layer, in contrast to present-day Mars (*13*, *14*) or the Moon (*16*). This difference in planetary structure and evolution is readily explained by their tectonic history. Proposed early-Earth tectonic regimes involve a heat pipe (*44*) or plutonic-squishy lid (*42*, *43*), and/or at least later on, plate tectonics (*45*, *46*). All these regimes are characterized by efficient crustal recycling, promoting reactive BMO crystallization. Similarly, proposed tectonic regimes for (early) Venus involve efficient crustal recycling (*83*), implying an Earth-like fate for any Venusian BMO [see ref. (*84*)]. In turn, Mars and the Moon have been governed by a stagnant lid with only minor crustal recycling (*85*, *86*), reconciling the long-term survival of a deep (partially) molten layer (*13*, *14*, *16*). Thus, the deep interior structure of terrestrial planets may help to constrain their magmatic and tectonic history, with implications for the magnetic field, atmospheric evolution, and habitability.

## MATERIALS AND METHODS

### BMO crystallization model

We use the thermodynamic model of ref. (*19*) to predict melt and solid compositions in equilibrium, as well as the relevant crystal fractions, during fractional and reactive crystallization in the lower mantle. For fractional crystallization, we incrementally remove solid compositions that are in chemical equilibrium with the coexisting liquid (BMO) compositions and update the liquid composition accordingly. To calculate FeO and MgO partitioning, we consider mineral-melt equilibrium constants of *K_bm_* = 0.1 for bridgmanite and *K_fp_* = 0.9 for ferropericlase. These values are calibrated for the ternary MgO-SiO_2_-FeO system (*19*).

For “reactive crystallization,” we consider coupled crustal addition, BMO cooling, and crystallization. In each incremental step, we first add crustal materials to the BMO, according to the crustal mass flux into the BMO, Φ. For model times *t* < 2.55 billion years (Gyr), 2.55 ≤ *t* < 4.209 Gyr, and *t* ≥ 4.209 Gyr, we add “Archean Basalt,” “Proterozoic Basalt,” and MORB compositions, respectively (see table S3). The former two compositions are based on ref. (*51*). As we assume perfect mixing between the added crustal material and the BMO, this addition results in an updated BMO composition. On the basis of this updated BMO composition as well as the updated BMO temperature (as constrained by our cooling curves in fig. S1; see Supplementary Results for details), we then calculate the new melt and solid compositions (and crystal fractions) using the same thermodynamic model as for fractional crystallization (*19*). We then proceed to the next incremental step.

Because of coupled cooling and addition during “reactive crystallization,” the crystal fraction is >0% in all incremental steps. In most of our cases, we consider *K_bm_* = 0.1 and *K_fp_* = 0.9, consistent with the thermodynamic model (*19*), but we also explore cases with different *K_bm_* and *K_fp_* (for details, see Supplementary Results and fig. S6). The nondimensional crustal addition rate, ψ = Φ/*m_BMO.init_* (with *m_BMO.init_* the initial mass of the BMO), is the main parameter to control the evolution of the BMO because there is a direct trade-off between Φ and *m_BMO.init_* or, likewise, between Φ and initial BMO thickness (Supplementary Results and table S1). In most but not all cases (fig. S2), we consider constant crustal addition rates (i.e., constant Φ and ψ) over time. We also explore the effects of different initial compositions of the BMO (table S3) and BMO cooling histories. Table S2 provides the range of parameters explored and our reference values.

### Model cases explored

For our main cases with reactive crystallization and FeO disproportionation switched on, we vary only one parameter at the time, keeping all other parameters fixed at reference values (table S2). This gives 16 cases with fixed *K_bm_* = 0.1 and *K_fp_* = 0.9 (table S2). As discussed in Supplementary Results and shown in fig. S6, we also run four more cases with *K_D_* = *K_bm_*/*K_fp_* = 0.6. We further run a few additional cases with other parameter combinations (but within the range shown in table S2), which are explicitly mentioned in the text when discussed. Then, we run several cases without FeO disproportionation (notably with variable *T_final_*), but only the case with reference values is shown (Figs. 2 and 4) and discussed here. Last, we run four cases with fractional crystallization (Fig. 2, red and pink lines). These cases are independent of (early) crustal addition rate and cooling history (we confirm that conclusions remain robust by running fractional-crystallization cases with *K_D_* = 0.6, analogous to those in fig. S6).

### FeO disproportionation

In natural Al-bearing systems, Al is incorporated into bm (*87*), driving FeO disproportionation (*38*, *39*, *53*). As Al_2_O_3_ is not included in the thermodynamic model applied here (*19*), we cannot account for its incorporation into bm explicitly. However, experimental work (although at shallow lower-mantle pressures) shows that the topology of the MgO-FeO-SiO_2_ ternary cross section through the phase diagram (*33*) is similar than that of the true ternary in ref. (*19*). In other words, the effects of Al incorporation into bm on the shape of the liquidus surfaces and notably on the locations of the cotectic valleys is rather minor.

Accordingly, we account for the effects of FeO disproportionation in a simplified way. We neglect the effects of Al incorporation into bm on the thermodynamic model, cumulate compositions and densities. We exclusively account for the related effects on the reduction of Fe^2+^O in the liquid BMO to Fe^0^ (i.e., through the disproportionation reaction) and removal of Fe^0^ from the BMO into the core. The incorporation of any 2 mol of AlO_1.5_ into bm as reduces 1 mol of FeO (in the liquid BMO) to Fe^0^. We assume that FeAlO_3_ is the preferred Al-bearing speciation of bm (*39*) and that all Fe in the initial BMO is present as ferrous iron (at the iron-wüstite buffer). We further assume a bm-melt partition coefficient for Al of 1.0 (*87*); FeO disproportionation is driven as long as Fe and Al are incorporated into bm together to stabilize FeAlO_3_. Any excess Al (or excess Fe) incorporated into bm does not drive disproportionation in our model.

### Density calculations

We calculate the densities of crystal cumulates from their MgO-SiO_2_-FeO compositions. In either cases with and without FeO disproportionation, we neglect Al incorporation into bm in terms of calculating densities for consistency with the thermodynamic model. The densities of all relevant mineral components (MgSiO_3_, FeSiO_3_, MgO, FeO, and SiO_2_) are calculated from a Mie-Grüneisen-Debye equation of state (*88*). The details of this calculation and a comparison of the density model with experimental constraints are provided in the Supplementary Materials (text S6 and table S4). The resulting densities of mineral endmembers at pressures of 130 GPa (and reference temperatures of 4000 K), from which all whole-rock cumulate density profiles are calculated (e.g., Figs. 2, 4, and 5), are reported in table S5. Densities of the liquid BMO are not explicitly calculated. We assume that they are always higher than the corresponding solid cumulates and the pyrolytic mantle.

### Pressure approximation

In all our calculations, including the BMO crystallization model and density calculations, we fix the pressure at 130 GPa as a simplified approximation. This approximation is appropriate as the liquid adiabat and liquidus (i.e., the crystallization temperatures) are near parallel over pressure in the lowermost mantle [e.g., ref. (*4*)]. BMO crystallization and chemical liquid-solid equilibration occurs near the top of the BMO (*2*). As the CMB pressure is ~135 GPa, the assumed pressure of 130 GPa is most appropriate for thin BMOs. For our reactive-crystallization cases, the BMO is relatively thin for most of its evolution. All our cases are characterized by fast initial solidification and an extended period, over which the BMO volume is rather small, i.e. ~10% of its initial volume (figs. S2A to S6A). For both reactive and fractional crystallization, the final stage of crystallization, which is most relevant for our main conclusions, is particularly characterized by thin BMOs. Test cases show that the typical cumulate density anomalies do not depend on our pressure approximation.

## Supplementary Materials

### This PDF file includes:

Supplementary Methods Supplementary Results Supplementary Discussion Figs. S1 to S10 Tables S1 to S5 References
