# Supplementary material for: Present-day Earth mantle structure set up by crustal pollution of the basal magma ocean
Source: Sci Adv. 2025 Jul 18;11(29):eadu2072. doi: 10.1126/sciadv.adu2072 (PMC12273756; doi:10.1126/sciadv.adu2072)
Supplement: Supplementary file 1 — Supplementary Methods Supplementary Results Supplementary Discussion Figs. S1 to S10 Tables S1 to S5 References [file sciadv.adu2072_sm.pdf]

Science Advances
AAAS

Supplementary Materials for

Present-day Earth mantle structure set up by crustal pollution of the basal magma ocean

Maxim D. Ballmer *et al.*

Corresponding author: Maxim D. Ballmer, m.ballmer@ucl.ac.uk

Sci. Adv. **11**, eadu2072 (2025)
DOI: 10.1126/sciadv.adu2072

This PDF file includes:

Supplementary Methods
Supplementary Results
Supplementary Discussion
Figs. S1 to S10
Tables S1 to S5
References

Supplementary Methods

Details of density calculations

We estimate lower-mantle density by considering mantle rocks composed of five components, namely FeO, MgO, SiO_2, $MgSiO_3$ and $FeSiO_3$. The two first components (FeO and MgO) enter into ferropericlase, the two last components ($MgSiO_3$ and $FeSiO_3$) enter into bridgmanite; SiO_2 forms a $CaCl_2$-type mineral. The density (ρ) of each component is calculated at a given pressure P and temperature T using the Mie-Grüneisen-Debye equation of state [88],

$$P = \frac{3K_{T0}}{2}\left[\left(\frac{\rho}{\rho_0}\right)^{7/3} - \left(\frac{\rho}{\rho_0}\right)^{5/3}\right] \left\{1 - \frac{3}{4}(4 - K'_{T0})\left[\left(\frac{\rho}{\rho_0}\right)^{2/3} - 1\right]\right\} + \Delta P_{th}, \tag{S1}$$

where the subscript zero indicates ambient conditions for density (ρ_0), isothermal bulk modulus K_{T0} and its pressure derivative K'_{T0}, while

$$\Delta P_{th} = \frac{\gamma(\rho)}{V}\left[E_{th}(\rho, T) - E_{th}(\rho, T_0)\right] \tag{S2}$$

is the thermal pressure with

$$V = \frac{\rho_0}{\rho} V_0 \tag{S3}$$

the volume (note that $\rho_0 = 1000M/V_0$ with M the molar mass),

$$\gamma = \gamma_0 \left(\frac{\rho_0}{\rho}\right)^q \tag{S4}$$

the Grüneisen parameter (q is a constant parameter),

$$E_{th} = \frac{9nRT^4}{\theta^3}\int_0^{\theta/T} \frac{x^3}{e^x - 1}\mathrm{d}x, \tag{S5}$$

the vibrational energy (calculated from the Debye model), R is the gas constant, n the number of atoms per formula unit, and

$$\theta = \theta_0\, e^{\frac{\gamma_0 - \gamma}{q}}, \tag{S6}$$

the Debye temperature. Solving equation S1 provides the density of each component at a given pressure and temperature. The density of the rock assemblage is then calculated considering a

simple arithmetic mean. All properties of the model are listed in table S4. For the purpose of this study, we consider a fixed pressure $P = 130$ GPa and temperature $T = 4000$ K (Materials and Methods; table S5), but the density model is tuned and tested for a wider range of P-T.

We test our density model by comparison with experimental results (see Figures S7 to S10). Particular emphasis has been placed on iron-rich compositions as they are critical for magma-ocean solidification and cumulate entrainment, notably in terms of dense late-stage cumulates, which are critical for our main conclusions. Overall, the predictions agree well with experimental results, particularly at the high pressures considered here. We note that for SiO_2 or for ferropericlase, the theoretical predictions agree well with some studies, while disagreeing with others. This discrepancy is explained as experimental results at such challenging physical conditions are somewhat sensitive to the experimental setup and/or techniques. It is therefore impossible to perfectly match all experimental results. Regardless, we emphasize that our density model is consistent with experimental data within the typical uncertainty between studies.

Supplementary Results

Effects of BMO initial size and crustal addition rate on BMO reactive crystallization

The effects of initial BMO size and crustal addition rate on reactive crystallization largely trade off with each other. The combined effects of both parameters can be summarized by investigating the relative crustal addition rate, ψ. The parameter ψ is the mass flux of basaltic crust into the BMO, Φ, divided by the initial mass of the BMO. In most of our cases, we assume constant ψ over time (but see fig. S3). Assuming crustal addition rates, Φ, that correspond to present-day subduction fluxes, the parameter ψ implicitly fixes the initial mass of the BMO, and hence its volume or initial thickness, $h_{BMO.init}$. The average Phanerozoic subduction flux is ~3.5 km^2/yr or ~24.5 km^3/yr [89, 90], corresponding to a mass flux of ~2.26×10^6 kg/s.

However, it is not obvious that the entire subducted basalt would segregate and settle at the base of the mantle to interact with the BMO. Also, subduction fluxes are largely uncertain before the Phanerozoic. Hence, particularly for BMOs that solidified well before the Phanerozoic, the relevant addition rates of basaltic materials are poorly known. Similarly, the initial thickness of the BMO is not well constrained (see main text). By varying ψ between 0.075 /Gyr and 0.5 /Gyr, we implicitly

explore these uncertainties and the coupled effects of initial BMO mass (or thickness, $h_{BMO.init}$) and crustal addition rate (or mass flux of basaltic material into the BMO, Φ). Table S1 provides the pairs of $h_{BMO.init}$ and Φ that cornenspond to a given value of ψ.

Figure S4 shows the effects of ψ on BMO reactive crystallization. Increasing ψ only slightly increases the lifetime of the BMO (fig. S4A). Hence, the BMO cools to slightly lower temperatures (fig. S1) for the given T_{final} = 3850 K and reaches slightly more enriched compositions (fig. S4B). The final-stage cumulates accordingly also reach slightly higher enrichment in iron, and slightly higher densities. In any case, these enrichments and densities remain in a moderate range (fig. S4C-D). More importantly, the total cumulate sequence tends to be much thicker (or more voluminous) for higher ψ, at least as long higher ψ reflect higher initial BMO volumes. Particularly the buoyant and the ~neutrally buoyant parts of the cumulate sequence with high bm contents notably increases in volume with increasing early addition rates. The volume of the intrinsically dense part of the cumulate sequence remains mostly robust for $h_{BMO.init} \leq 500$ km.

The efficiency of FeO disproportionation and average oxidation of the cumulate package also depend on ψ. Average $Fe^{3+}/\Sigma Fe$ increases with increasing ψ, i.e., from ~42% to ~72% for the cases shown in figure S4. Accordingly, average $Fe^{3+}/\Sigma Fe$ tends to decrease with increasing initial BMO thickness.

Effects of early crustal addition rate

We also explore the effects of early crustal addition rate on BMO reactive crystallization. In these cases, the early addition rate (i.e., at ¡2.25 Gyr or equivalently at ¿2.3 Ga) is different from the late addition rate (i.e., at ¿2.25 Gyr or equivalently at ¡2.3 Ga), ψ. In other words, the crustal addition rate is not constant over time. In the cases explored and shown in figure S2, the late addition rate is fixed at ψ = 0.224 /Gyr. Along with the reference case (i.e., with constant addition rate over time), cases with early addition rates of 0.112 /Gyr, 0.448 /Gyr and 0.896 /Gyr are shown in figure S2.

Increasing the early addition rate increases the size of the BMO in the Archean eon, and extends its total lifetime (fig. S2A). Hence, the BMO cools to lower temperatures (fig. S1) for the given T_{final} = 3850 K, and reaches more iron-enriched compositions (fig. S2B). Accordingly, the final-stage cumulates are also more enriched, and display higher densities. In any case, these enrichments and densities (up to ~5,800 kg/m^3) remain in a moderate range (fig. S2C-D). Importantly, the total

cumulate sequence is significantly thicker (or more voluminous) for higher early addition rates. The intrinsically dense part of the sequence (see Figure 5A), as well as the ~neutrally dense part with high bm contents, increases in volume with increasing early addition rates.

The efficiency of FeO disproportionation and average oxidation of the cumulate package also depend on early addition rate. Average $Fe^{3+}/\Sigma Fe$ increases with increasing early addition rate from ~50% to ~80% for the cases shown in figure S2. This result is explained by a stronger pollution of the BMO with Fe, Si and Al for higher addition rates, which drives disproportionation in bm.

Effects of BMO initial composition

We also investigate the effects of BMO initial composition on reactive crystallization. We consider different initial compositions, as they are relevant for different BMO formation scenarios. For example pyrolytic and Hadean-basaltic initial compositions (Fig. 1, black and purple circles) are relevant for the "density crossover" [2] and "overturn" [9] BMO formation scenarios, respectively (see main text). We consider Archean basaltic rocks [51] as a proxy for Hadean Basalt. The "intermediate" case is a 1:1 mix of pyrolite and Archean Basalt, representing a hybrid scenario, in which both a density crossover and a global-scale overturn contributed to BMO formation. For all the above three initial BMO compositions, the molar fractions of main oxides are reported in table S3.

Figure S5 shows that increasing the iron and silica contents in the initial BMO (i.e., from "pyrolite" to "intermediate" to "Archean Basalt") increases the size of the BMO in the Archean eon, and extends its total lifetime (fig. S5A). Hence, the BMO cools to lower temperatures (fig. S1) for the given T_{final} = 3850 K and reaches more iron-enriched compositions (fig. S2B). Accordingly, the final-stage cumulates are also more enriched, and display higher densities. In any case, these enrichments (up to Fe# ≈ 56) and densities (up to ~5,800 kg/m^3) remain in a moderate range (fig. S2C-D), i.e., much lower than those predicted by fractional crystallization (i.e., Fe# ≈ 100 and densities up to ~7,600 kg/m^3). The total volume of the cumulate sequence is similar for different BMO initial compositions, but that of the intrinsically dense part of the sequence is smaller for pyrolytic initial compositions than for the more iron/silica enriched BMO initial compositions (fig. S5C).

As basaltic/intermediate BMOs are initially relatively enriched in Si and Fe, and notably in

Al, FeO disproportionation and related oxidation of the mantle is further more efficient than for pyrolitic BMOs. For otherwise reference parameters, $Fe^{3+}/\Sigma Fe$ as averaged over the entire cumulate sequence is 89.2% for Archean-basaltic and 85.9% for intermediate initial BMO compositions. In turn, it is just 56.8% for pyrolotic initial compositions (i.e., for the reference case).

Effects of cooling history

As the BMO is liquid and assumed to be well mixed (i.e., not stably stratified), the cooling of the BMO and core are coupled. This coupled cooling history is based on ref. [91], a 2D geodynamic study of Earth evolution which includes a primordial basal layer. The effects of such a layer on core cooling are similar to those of a BMO. We parameterize core cooling based on two model runs (i.e., with friction coefficients of 0.02 and 0.08) in ref. [91]. We interpolate between these two geodynamically-predicted cooling curves in such a way that the CMB temperature after 4.55 Gyr reaches T_{final}. Proposed values for present-day Earth's CMB temperatures, T_{final}, range from ~3,500 to ~4,200 K [92, 36, 34, 35]. However, for the entire range of parameters considered here (i.e., for any combination of parameters within the range given in table S2), we find that a BMO with significant thickness survives until the present-day for T_{final} ≥3970 K, inconsistent with geophysical constraints for the Earth's mantle [18]. Thus, we explore T_{final} between 3500 K and 3950 K (reference case: T_{final} = 3850 K).

Figure S3 shows the effects of BMO cooling history on BMO reactive crystallization. Increasing T_{final} extends the lifetime of the BMO (fig. S3A), and therefore the total mass of crustal material added (over time) to the BMO (at a given Φ). This notably increases the total volume/mass of the cumulate sequence (fig. S3A). Nevertheless, the final iron enrichment of the BMO and of the corresponding cumulates remain largely robust (fig. S3A, D). Therefore, the maximum density anomaly reached at the base of the cumulate pile (i.e., ~250 kg/m^3 higher than the ambient mantle) is also largely independent of T_{final} (fig. S3C). Only the case with the highest T_{final} (i.e., 3950 K), which exhibits a BMO that survives until (and beyond) the Phanerozoic, predicts a cumulate sequence with density anomalies of only up to ~150 kg/m^3. These rather low density anomalies are explained by the addition of strongly silica-enriched materials (=MORB) in the Phanerozoic, which increases the stishovite fraction in, and therefore decreases the density of, late-stage cumulates.

The efficiency of FeO disproportionation and average oxidation of the cumulate package also

depend on BMO cooling history. Average $Fe^{3+}/\Sigma Fe$ increases with increasing T_{final} from 49.1% to 64.4% for the cases shown in figure S3. This result is explained by longer BMO lifetimes for higher T_{final}, which allows a stronger crustal pollution, particularly in the Phanerozoic (MORB is most enriched in Si and Al; see table S3).

Effects of mineral-melt partitioning

In most of our cases, including the reference case, we consider mineral-melt equilibrium constants K_{bm} = 0.1 for bridgmanite (bm) and K_{fp} = 0.9 for ferropericlase (fp), consistent with the thermodynamic model of ref. [19]. For each mineral (bm or fp), $K_{mineral}$ = (Fe#$_S$/Mg#$_S$) / (Fe#$_L$/Mg#$_L$) with Fe# = 1−Mg#, and subscripts S and L referring to the solid and liquid compositions in equilibrium, respectively. These two coefficients are linked by $K_D = K_{bm} / K_{fp}$. In ref. [19], these coefficients are calibrated for the ternary MgO-FeO-SiO_2 system. In such an Al-free system with the relevant K_{fp} = 0.9 and K_{bm} = 0.1, K_D = 0.11, i.e., iron is primarily taken up by fp. In Earth-like Al-bearing systems, however, it is more efficiently taken up by bm than in Al-free systems. Realistic Al-bearing systems are better described by K_D ≈0.6 [93, 94], notably because iron can also be incorporated as $FeAlO_3$ into bm, consistent with our cases that consider FeO disproportionation.

In order to consider these more realistic K_D, but also to account for experimental uncertainties, we run two additional cases. In the low-K case, we assume K_{bm} = 0.075 and K_{fp} = 0.125. In the high-K case, we assume K_{bm} = 0.105 and K_{fp} = 0.175. These ranges of $K_{mineral}$ bracket the results of experimental studies for Al-bearing systems at ~130 GPa. According to ref. [95], K_{bm} is 0.08~0.1, consistent with ref. [96], but note that both these studies report lower-mantle iron partitioning in different notations [19]. In turn, K_{fp} is constrained as ~0.1 at ~130 GPa [97], but there are only few experimental studies at these conditions. Both our low-K and high-K cases imply $K_D = K_{bm} / K_{fp}$ = 0.6, consistent with various experimental studies [98, 94, 93].

Figure S6 shows the results for the low-K and the high-K cases. The lifetime of the BMO, the final iron enrichments of the BMO and of the corresponding cumulates, as well as the maximum cumulate densities are virtually the same for the high-K cased and for the corresponding case with reference values (fig. S6 vs. S4). This result is explained by the dominant effects of K_{bm}, which is very similar in both these cases (0.1 and 0.105). K_{bm} controls the melt-solid iron partitioning, and hence cumulate density, for most of the crystallization sequence (i.e., since fp only crystallizes

near the beginning of the sequence for pyrolitic initial compositions). Comparing the high-K and low-K cases highlights that the lifetime of the BMO tends to be higher for lower K_{bm} , because iron is less efficiently removed from the liquid BMO. Therefore, the BMO (as well as eventually the corresponding final-stage cumulates) reach higher iron enrichments and lower temperatures. Nevertheless, the maximum densities in the cumulate sequence remain mostly unaffected.

The most important effect of K_{fp} is to change the iron partitioning between fp and bm in the ambient mantle pyrolite. In other words, the density of the ambient-mantle pyrolite depends on $K_D = K_{bm} / K_{fp}$. For the high-K and low-K cases, $K_D = 0.6$ and the ambient-mantle pyrolite density is ~5450 kg/m^3 (at 130 GPa). For all other cases (including the reference case), $K_D \approx 0.11$ and the ambient-mantle density is ~5500 kg/m^3. It is almost exclusively this difference in ambient-mantle densities, which accounts for the higher density anomalies in the high-K and low-K cases compared to the corresponding cases with reference values. Nevertheless, the density anomalies remain in a moderate range (up to ~300 kg/m^3), and our conclusions therefore remain robust.

The efficiency of FeO disproportionation and average oxidation of the cumulate package also depend on partitioning coefficients, particularly on K_D. Average $Fe^{3+}/\Sigma Fe$ of the high-K and low-K cases, which both have $K_D = 0.6$, are similar (at otherwise the same parameters), varying just within ~5%. However, they are significantly higher (i.e., by ~20%) than corresponding cases with $K_D \approx 0.11$ (i.e., as most of our cases. This result is explained by less efficient incorporation of Fe into fp during the early stage of BMO crystallization, and thus a higher availability of Fe for incorporation into bm, for high $K_D = 0.6$. Similarly, the mass of disproportionated Fe added to the core increases with K_D. For K_D=0.6 and ψ=0.224, this mass is 16%~18% of that of the inner core (vs. ~14% for the reference case), and for K_D=0.6 and ψ=0.15, it is 19%~22% of that of the inner core.

Supplementary Discussion

BMO cumulate entrainment

Late-stage BMO reactive-crystallization cumulates are a good candidate material for LLVPs. The intrinsically dense part of the cumulate sequence ranges from density anomalies of ~50 kg/m^3 to ~200 kg/m^3 or up to ~300 kg/m^3, depending on parameters (Figs. 5, S2-S6). These density

anomalies correspond to average density anomalies of ~125 kg/m^3 to ~175 kg/m^3, assuming internal mixing within piles. For inefficient internal mixing due to high intrinsic viscosities [99], the relevant density anomalies of the lowermost cumulate layer would be somewhat higher. Such density anomalies are within the range of materials that typically form long-lived thermochemical piles in geodynamic models. Ref. [22] find that buoyancy numbers of 0.25~0.35 lead to long-lived piles with small entrainment rates. These buoyancy numbers, which quantify the ratio between negative compositional and positive thermal density anomalies, correspond to density anomalies of ~150 kg/m^3 to ~250 kg/m^3 [22]. Other geodynamic studies agree with this result [20, 21], finding upper-bound buoyancy numbers of ~0.4 for a thermochemical-pile regime (i.e., higher values lead to stable global layer regime). In a different setup, ref. [23] find a thermochemical layer with significant topography (but mostly no isolated piles) for buoyancy numbers of 0.6~0.7. The tendency for intrinsically dense material to form piles also depends on their temperature and viscosity contrast with respect to the ambient mantle [100, 99]. Indeed, ref. [100] finds thermochemical piles for buoyancy numbers up to ~0.9 for excess internal heating within the piles.

In any case, geodynamic studies have repeatedly highlighted the main control of intrinsic density anomalies for an "LLVP-like" thermochemical pile-regime [20, 23, 24, 91, 22, 99, 101, 100]. For different choices of thermal expansivity (1×10^{-5} K^{-1} to 2×10^{-5} K^{-1}), the range of relevant buoyancy numbers in the literature (0.25~0.8, reported above) corresponds to density anomalies of ~50 kg/m^3 to ~300 kg/m^3. This density range is visualized in Figure 2 (yellow bar), and remains a conservative estimate, since the thermal expansivity in the lowermost mantle is likely well below 2×10^{-5} K^{-1} [102].

These geodynamically constrained material properties for LLVP candidate materials agree well with cumulate densities due to BMO reactive crystallization: For part 3 of the cumulate sequence (i.e., as defined in the main text; see Fig. 4), we predict average density anomalies of 100~200 kg/m^3 and peak density anomalies of 150~300 kg/m^3, depending on case (Figures 2, 5, S2C-S6C). In contrast, maximum density anomalies of BMO fractional-crystallization cumulates exceed 2000 kg/m^3, and corresponding buoyancy numbers exceed ~10, well within the global layer regime.

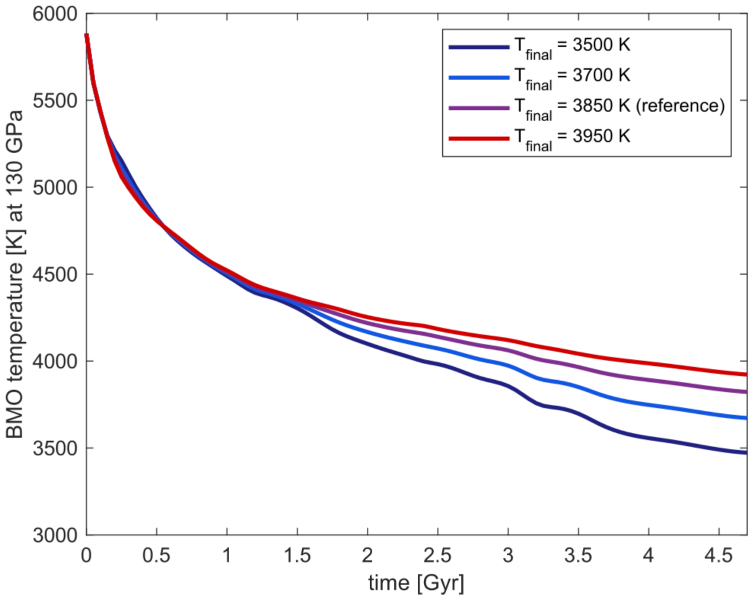

Figure S1: **Cooling histories of the BMO at the reference pressure of 130 GPa for different present-day (4.55 Gyr) CMB temperatures,** T_{final}. These BMO cooling histories are based on core cooling histories from ref. [91]. CMB temperatures (i.e., at 135 GPa) are projected to 130 GPa using adiabats according to ref. [91].

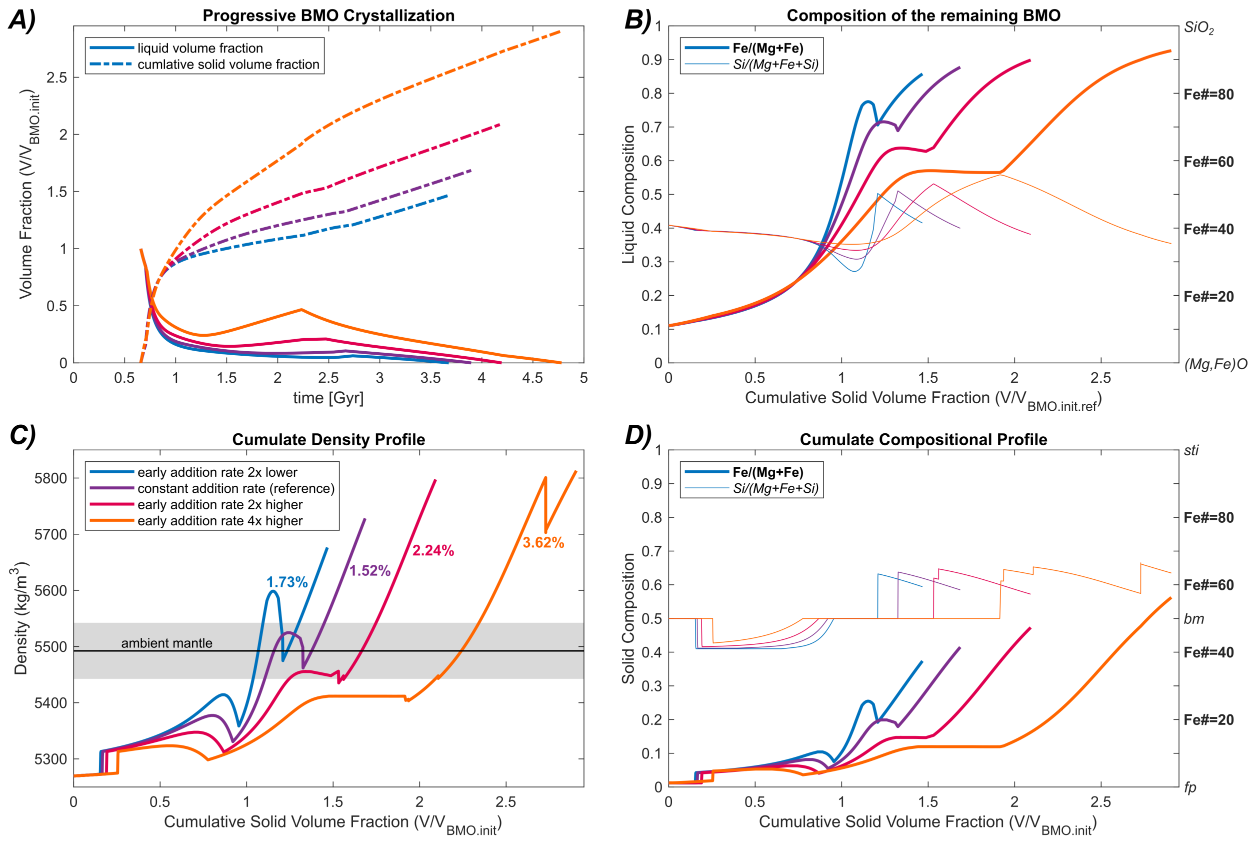

Figure S2: **BMO reactive crystallization as a function of early addition rate**. Progression of BMO crystallization over time (A), compositional evolution of the BMO during crystallization (B), and cumulate density (C) and compositional (D) profiles. The early addition rate is labeled in panel (C). The early addition rate (i.e., <2.25 Gyr or >2.3 Ga) is varied between 0.5x and 4x that of the late addition rate (i.e., >2.25 Gyr or <2.3 Ga). The late addition rate is fixed at ψ = 0.224 /Gyr, which corresponds to Phanerozoic subduction fluxes of 24.5 km^3/yr [89, 90] assuming an initial BMO thickness of 350 km (also see table S1). All other parameters as in the reference case (see table S2). Cumulate densities are calculated at 130 GPa and 4000 K (see Materials and Methods). Colored annotations and grey bar in (C) as in Figure 5 (main text).

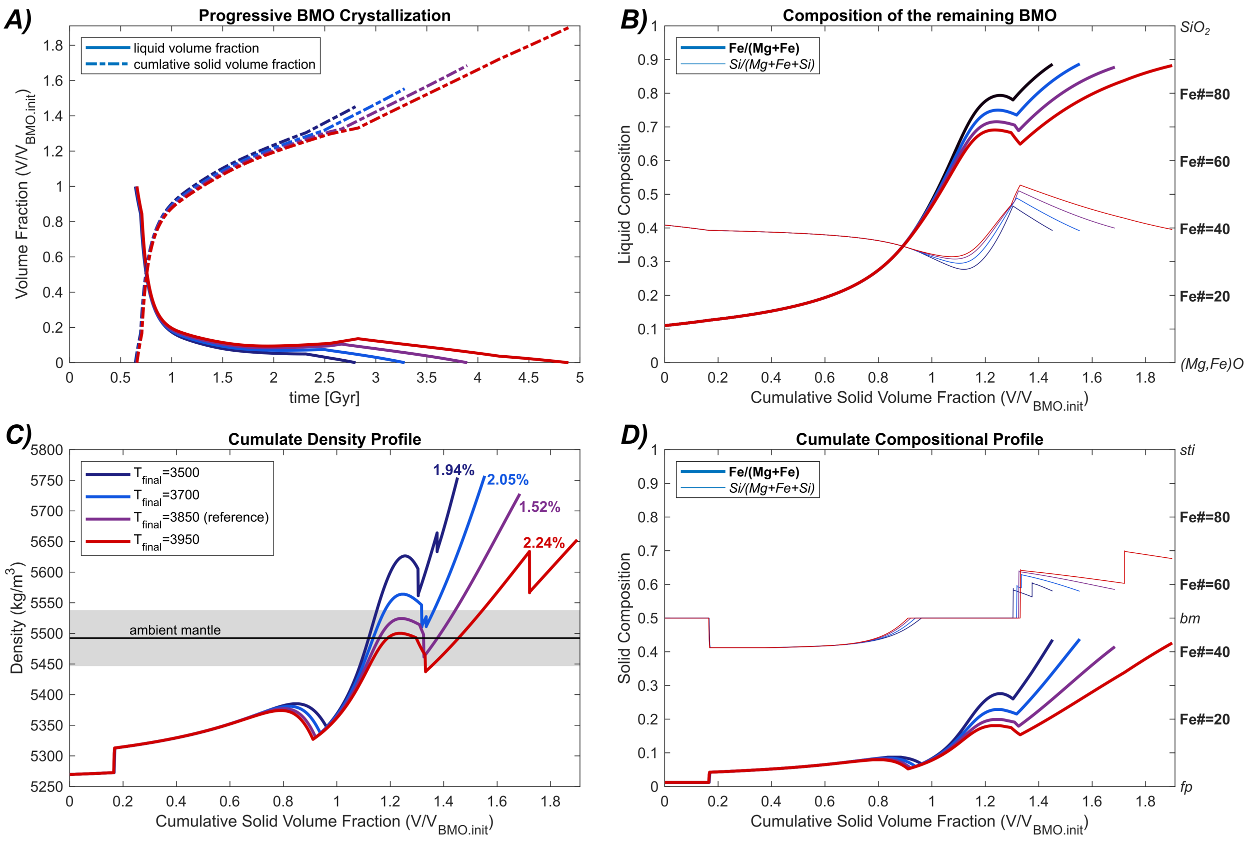

Figure S3: **BMO reactive crystallization as a function of present-day CMB temperature**. Progression of BMO crystallization over time (A), compositional evolution of the BMO during crystallization (B), and cumulate density (C) and compositional (D) profiles. The assumed present-day (4.55 Gyr) CMB temperature, T_{final} is labelled in panel (C). All other parameters as in the reference case (see table S2). The BMO cooling history as a function of T_{final} is shown in fig. S1. Cumulate densities are calculated at 130 GPa and 4000 K (see Materials and Methods). Colored annotations and grey bar in (C) as in Figure 5 (main text).

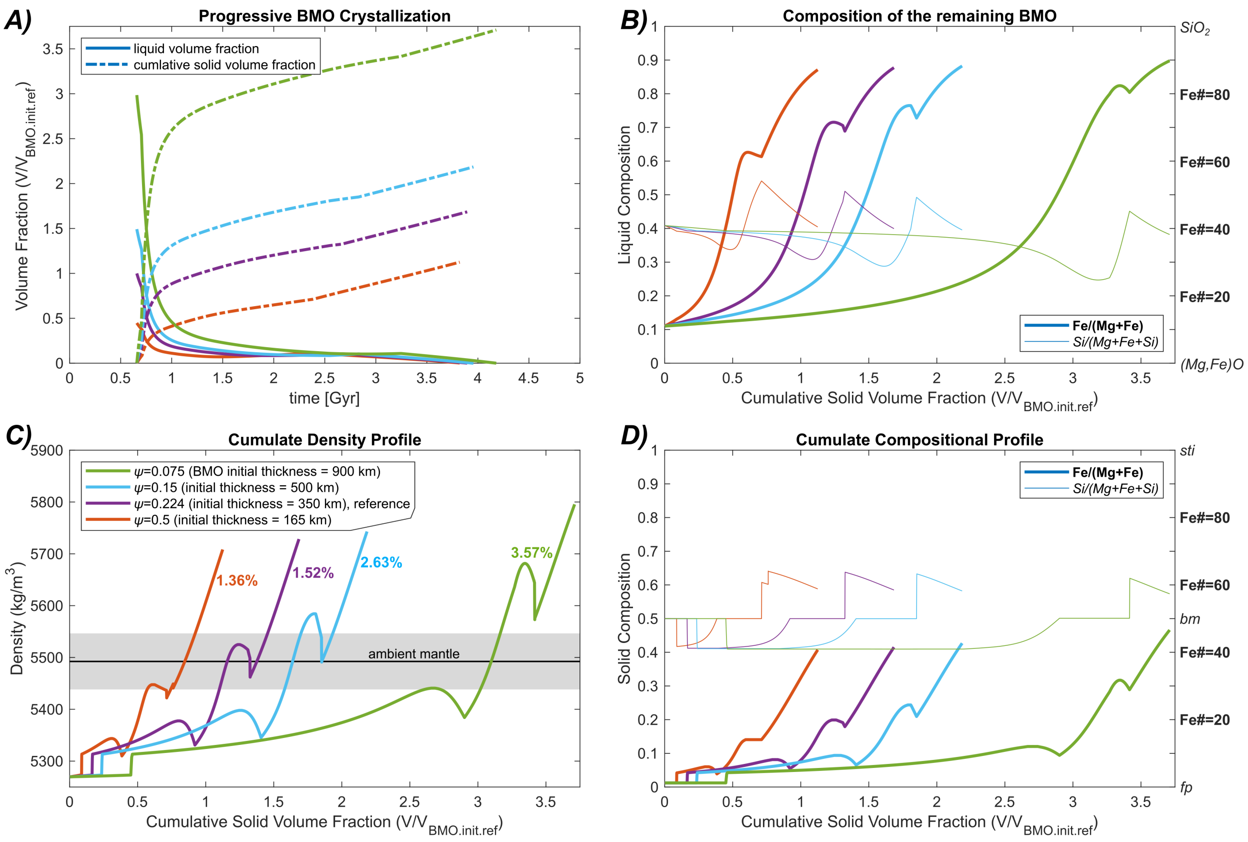

Figure S4: **BMO reactive crystallization as a function of addition rate**. Progression of BMO crystallization over time (A), compositional evolution of the BMO during crystallization (B), and cumulate density (C) and compositional (D) profiles as a function of the non-dimensional addition rate, ψ. The non-dimensional addition rate, ψ, is the initial mass of the BMO divided by the mass flux of basaltic material into the BMO, Φ. Assuming $\Phi = 2.26\times10^6$ kg/s (which corresponds to the average Phanerozoic subduction flux, see above), the curves shown can be understood as representing cases with different initial BMO thickness, $h_{BMO.init}$ (Note that the volume fractions in the Figure are scaled accordingly; i.e., such that lower ψ imply proportionately higher initial BMO volumes). For trade-offs between $h_{BMO.init}$ and Φ in terms of the relevant values of ψ, see table S1. All other parameters as in the reference case (see table S2). Cumulate densities are calculated at 130 GPa and 4000 K (see Materials and Methods). Colored annotations and grey bar in (C) as in Figure 5 (main text).

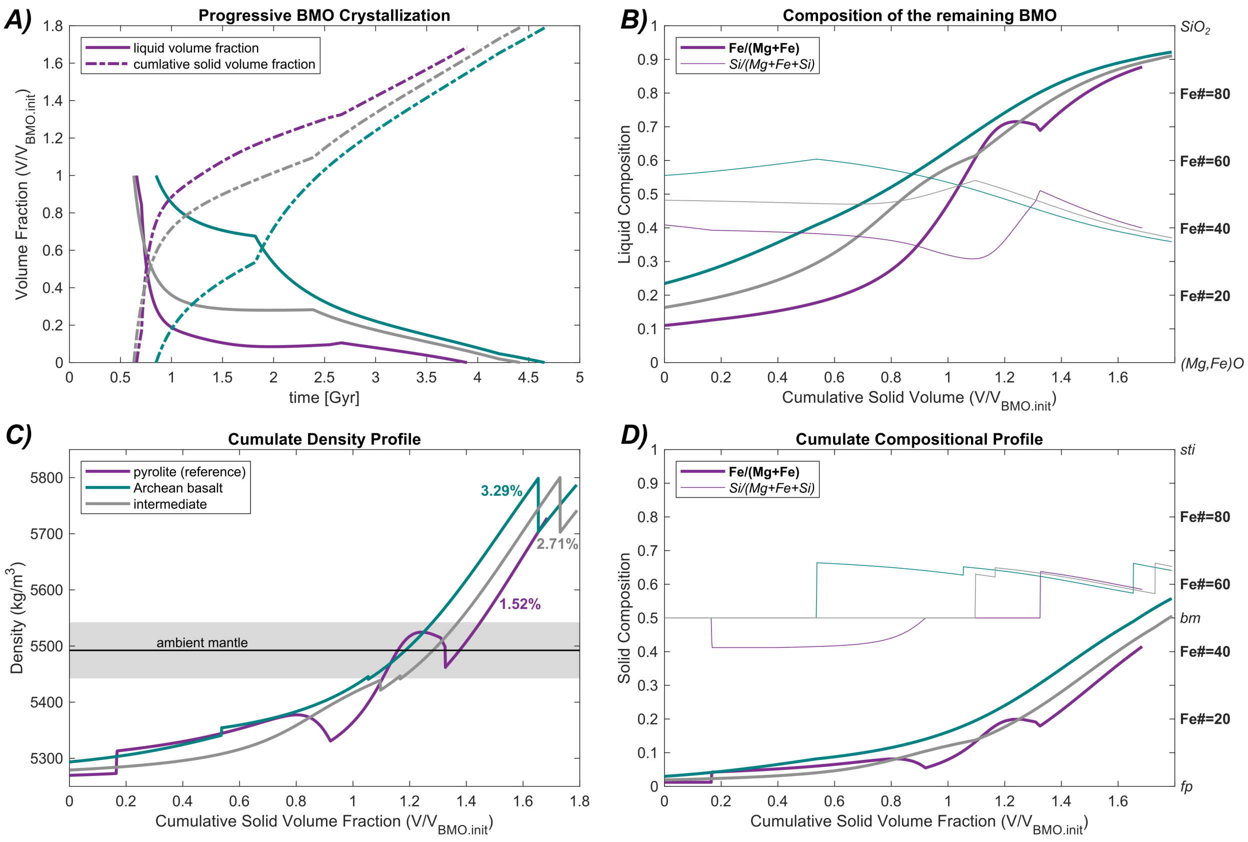

Figure S5: **BMO reactive crystallization as a function of initial composition**. Progression of BMO crystallization over time (A), compositional evolution of the BMO during crystallization (B), and cumulate density (C) and compositional (D) profiles for different BMO initial compositions (as labelled in panel (C)). All other parameters as in the reference case (see table S2). Archean basalt is a proxy for Hadean basalt. The MgO-SiO_2-FeO-Al_2O_3 contents for all BMO initial compositions considered are provided in table S3. Cumulate densities are calculated at 130 GPa and 4000 K (see Materials and Methods). Colored annotations and grey bar in (C) as in Figure 5 (main text).

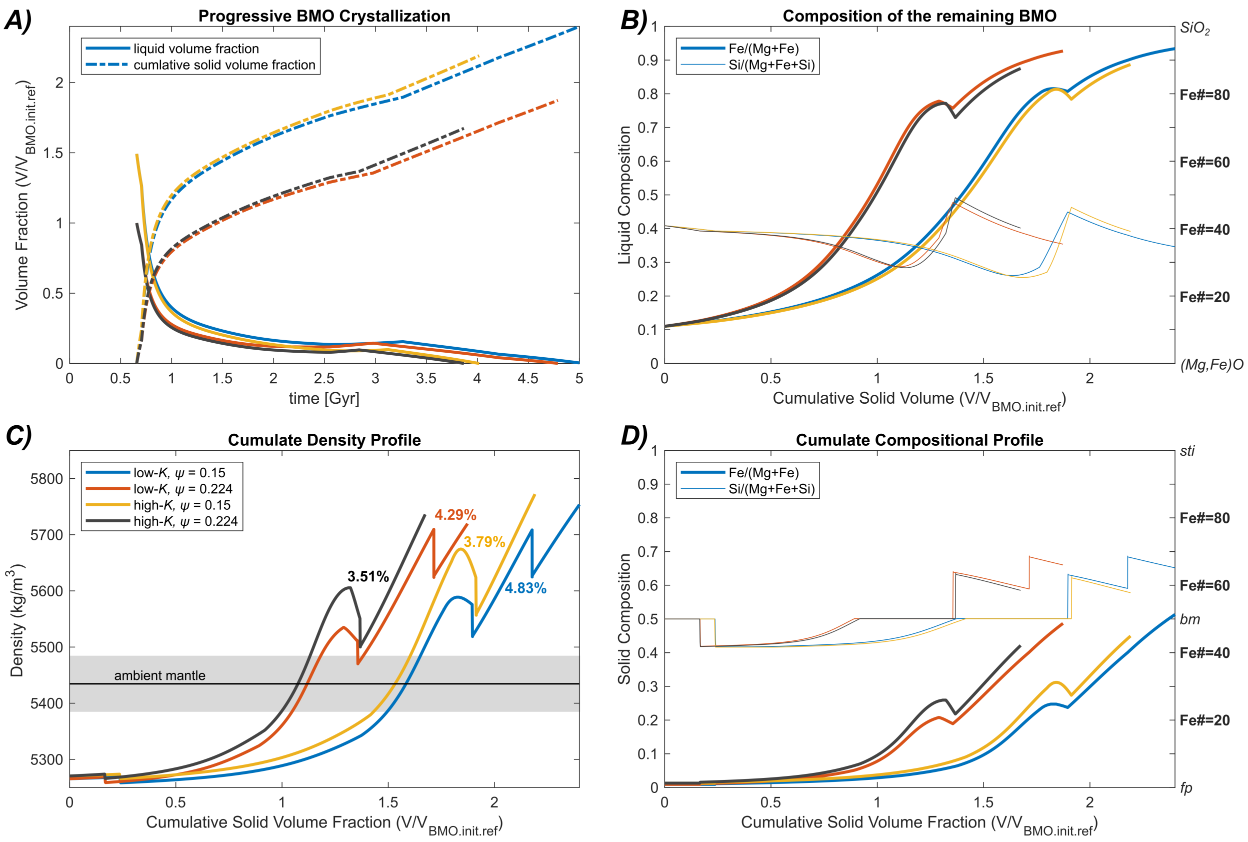

Figure S6: **BMO reactive crystallization as a function of mineral-melt partitioning and addition rate**. Progression of BMO crystallization over time (A), compositional evolution of the BMO during crystallization (B), and cumulate density (C) and compositional (D) profiles for different equilibrium constants, and different ψ (as labelled in panel (C)). Colored annotations and grey bar in (C) as in Figure 5 (main text). The low-K cases have equilibrium constants of K_{bm} = 0.075 and K_{fp} = 0.125. The high-K cases have K_{bm} = 0.105 and K_{fp} = 0.175. All other parameters as in the reference case (see table S2). Cumulate densities are calculated at 130 GPa and 4000 K (see Materials and Methods).

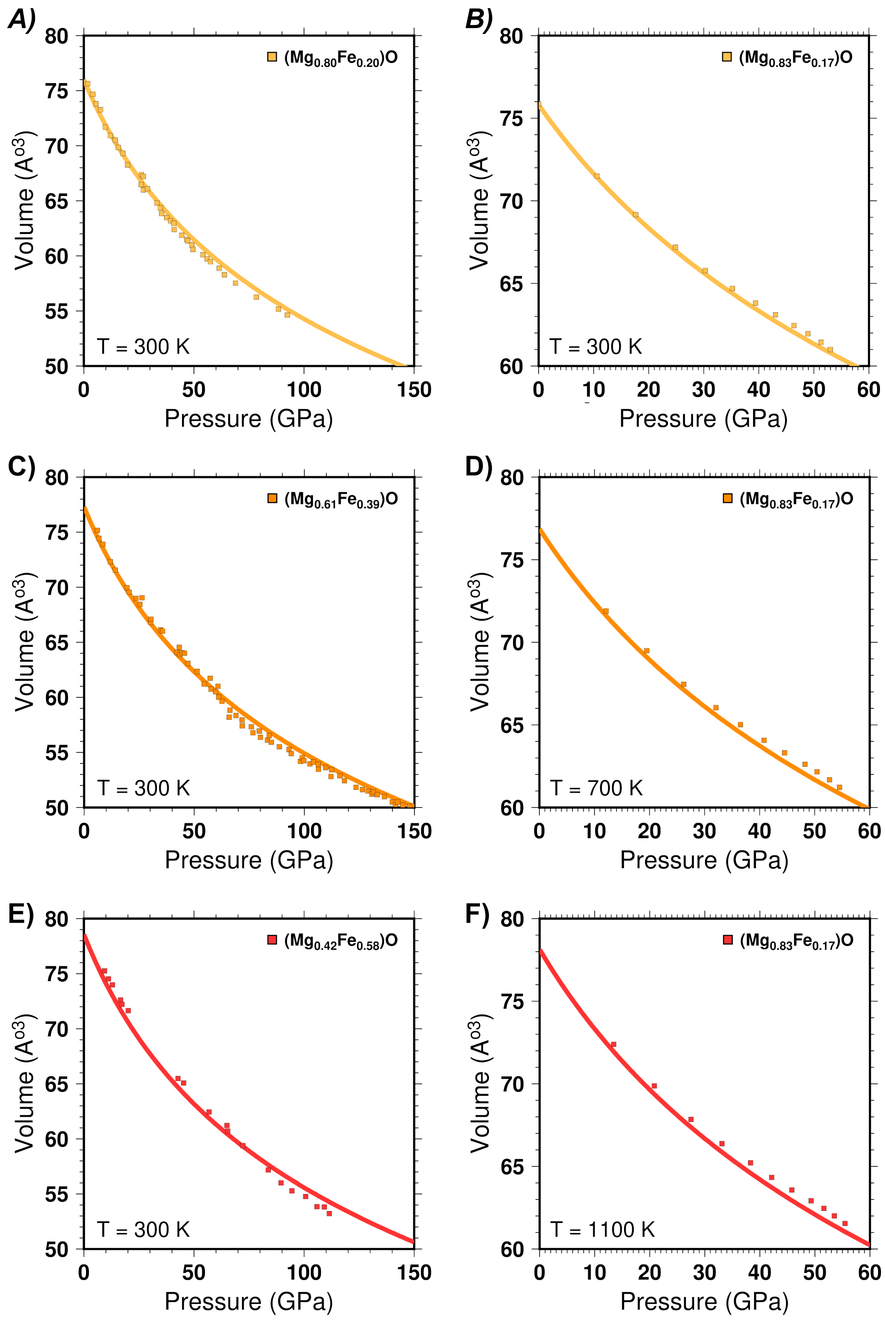

Figure S7: **Test of the ferropericlase density model.** Comparison of theoretical predictions (solid lines) with experimental data (square symbols) of the volume of ferropericlase for various FeO contents (as labelled) and temperatures. (A,C,E) Comparison with experimental data from ref. [103] at T = 300 K for three different fp compositions: with (A) Fe# = 20, (C) Fe# = 39, (E) Fe# = 58. (B,D,F) Comparison with experimental data from ref. [104] for a fixed fp composition (with Fe #= 17) at three different temperatures: (B) T = 300 K, (D) T = 700 K, (F) T = 1100 K.

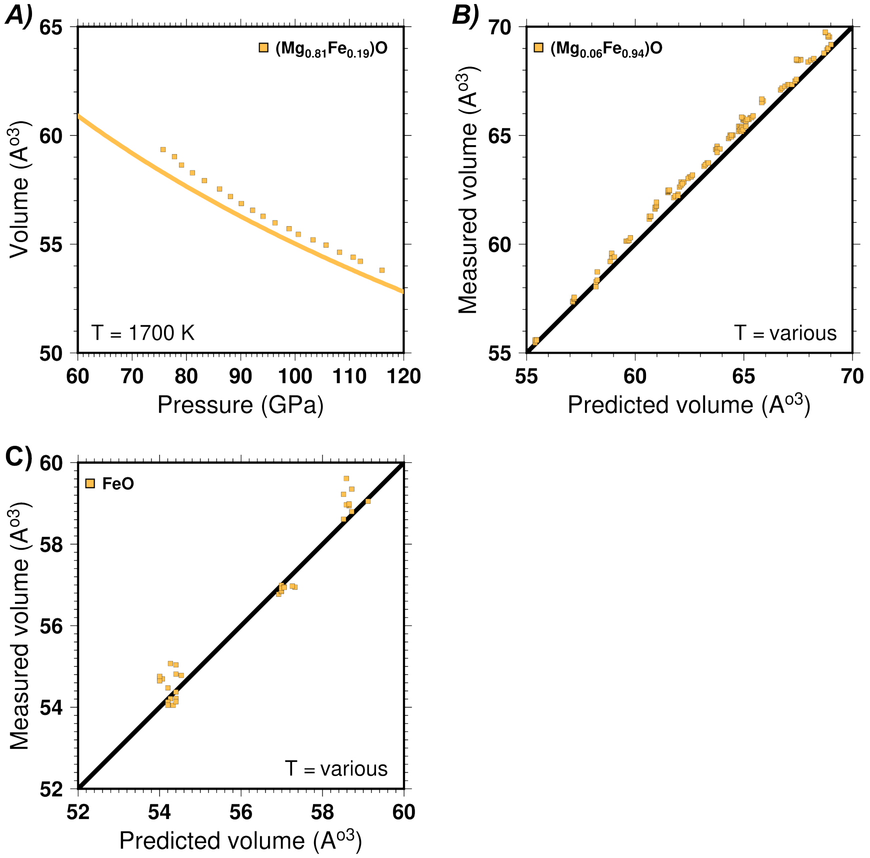

Figure S8: **Test of the ferropericlase density model (continued).** Comparison of theoretical predictions (solid lines) with experimental data (square symbols) of the volume of ferropericlase for various FeO contents (as labelled) and temperatures. (A) Comparison with experimental data from ref. [105] at T = 1700 K (fp composition as labelled). (B) Comparison with experimental data from ref. [25] at various temperatures (fp composition as labelled). (C) Comparison with experimental data for FeO from ref. [106] at various temperatures.

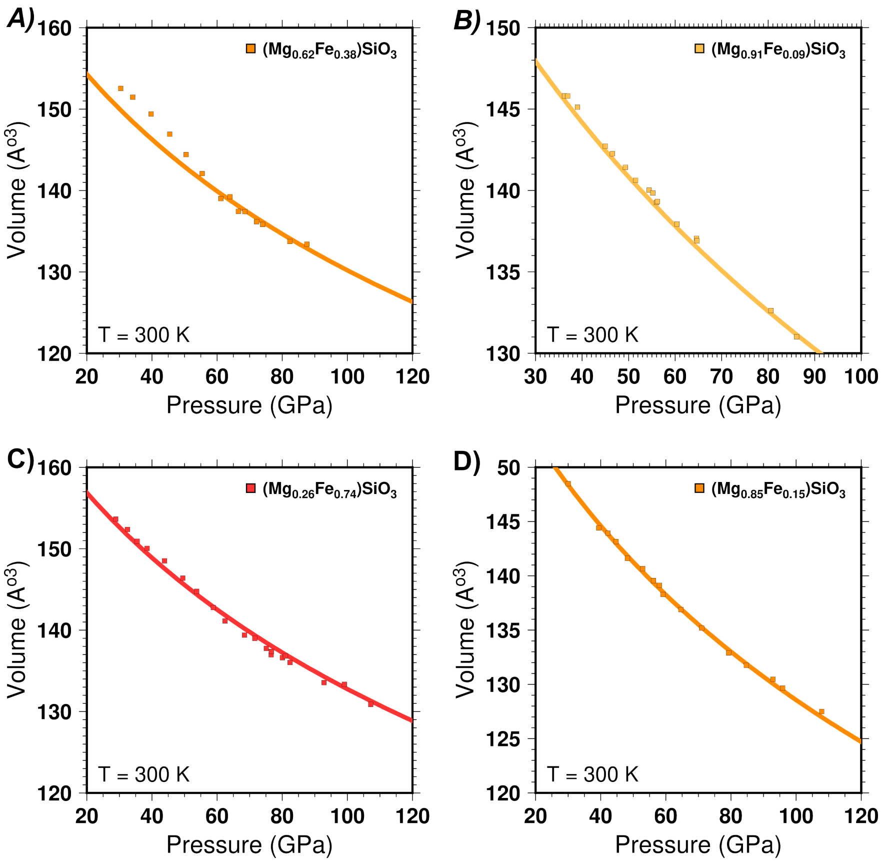

Figure S9: **Test of the bridgmanite density model.** Comparison of theoretical predictions (solid lines) with experimental data (square symbols) of the volume of bridgmanite for various FeO contents and temperatures. (A,C) Comparison with experimental results from ref. [107] at T = 300 K for two different FeO contents of bm: with (A) Fe# = 38, (C) Fe# = 74. (B,D) Comparison with experimental results from ref. [108] at T = 300 K for for two different FeO contents of bm: with (B) Fe# = 9, (D) Fe# = 15.

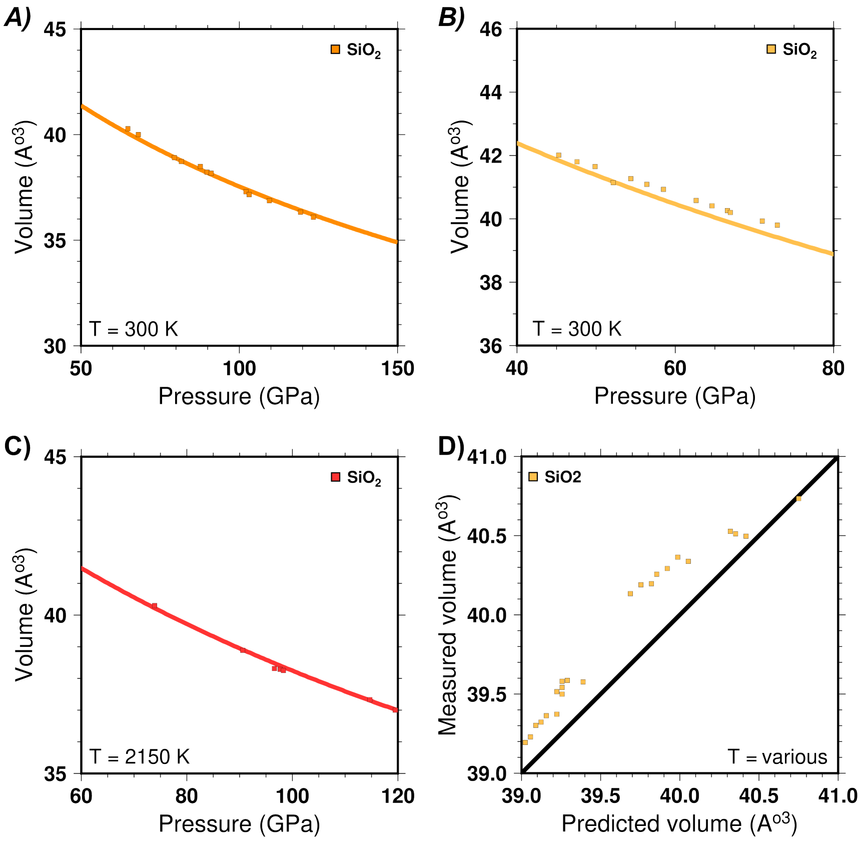

Figure S10: **Test of the stishovite density model.** Comparison of theoretical predictions (solid lines) with experimental data (square symbols) of the volume of $CaCl_2$-type SiO_2 at various temperatures. (A) Comparison with experimental results from ref. [109] obtained at T = 300 K. (B) Comparison with experimental results from ref. [110] obtained at variable temperatures. (C) Comparison with experimental results from ref. [109] obtained at T = 2150 K. (D) Comparison with experimental results from ref. [111] obtained at variable temperatures.

Table S1: **BMO initial thickness, as a function of ψ and Φ.** The parameter varied in our models is the non-dimensional addition rate of basaltic material, ψ. For a given mass flux of basaltic material into the BMO, Φ (columns), any ψ value (rows) fixes a corresponding initial BMO mass and initial BMO thickness, $h_{BMO.init}$. The average Phanerozoic MORB subduction flux is $\Phi_0 = 2.26 \times 10^6$ kg s^{-1}.

ψ	$\Phi = \Phi_0 = 2.26 \times 10^6$ kg s^{-1}	$\Phi = 0.2 \times \Phi_0$	$\Phi = 0.5 \times \Phi_0$	$\Phi = 2 \times \Phi_0$	$\Phi = 4 \times \Phi_0$
0.075	~900 km	~217 km	~501 km	~1532 km	~2461 km
0.15	~500 km	~111 km	~267 km	~899 km	~1530 km
0.224	~350 km	~75.5 km	~183 km	~645 km	~1134 km
0.5	~165 km	~34.3 km	~84.4 km	~316 km	~587 km

Table S2: **Parameter values explored in our our study, and reference values.** Due to a direct trade-off, any value of ψ can be understood as various pairs of Φ and $h_{BMO.init}$ (see table S1). For most cases presented and discussed throughout the study, only a single parameter is varied at the time (with all other parameters fixed to reference values). K_D, K_{bm}, and K_{fp} are co-varied as discussed in the last subsection of the Supplementary Results. This gives twenty main cases, which are all visualized in Figures S2-S6. For details of all cases explored, see Methods section.

Parameter (unit)	Values explored	Reference value
ψ	0.075, 0.15, 0.224, 0.5	0.224
Φ_{early} (kg s^{-1})	0.5×Φ,Φ, 2×Φ, 4×Φ	Φ
T_{final} (K)	3500, 3700, 3850, 3950	3850
Initial BMO composition	Pyrolite, Archean Basalt, Intermediate	Pyrolite
$K_D = K_{bm}/K_{fp}$	0.111, 0.6	0.111
K_{bm}	0.075, 0.1, 0.105	0.1
K_{fp}	0.125, 0.175, 0.9	0.9

Table S3: **Molar fractions of major oxides for all three scenarios of BMO initial composition, and all three flavors of added crustal material**. Molar fractions are normalized by those of $MgO+SiO_2+FeO$. The assumed time range for addition of MORB is based on the beginning of the Phanerozoic eon at 0.541 Ga, considering a slab sinking time of 200 Myr [112, 113] The composition of Archean Basalt is an average of Archean and early Proterozoic (≥2 Ga) basaltic rocks from ref. [51]. There is a significant compositional difference between these groups of rocks (Figure 1b).

Compositions of the initial BMO[1] and added crustal material[2]	Time range of addition	MgO	SiO_2	FeO	Al_2O_3
Pyrolite[1]	-	0.52623	0.40864	0.06513	0.04687
Archean Basalt[1,2]	t <2.55 Gyr	0.3402	0.5554	0.1044	0.1218
Intermediate[1]	-	0.4332	0.4820	0.0848	0.0844
Proterozoic Basalt[2]	2.55≤ t <4.209 Gyr	0.2949	0.6056	0.0995	0.156
MORB[2]	t ≥4.209 Gyr	0.2026	0.7017	0.0957	0.2763

Table S4: **Equation of state parameters for all relevant mineral components**. These parameters are needed to solve equations S1-S6 for density as a function pressure, P and temperature, T. For notations, see Suppl. Text. The resulting densities of the five mineral components at P=130 GPa and T=4000 K, see table S5.

	Unit	MgO	FeO	SiO_2	$MgSiO_3$	$FeSiO_3$
K_{T0}	GPa	161[a]	149.4[b]	245[c]	251[a]	272[a]
K'_{T0}		3.8[a]	3.6[b]	4.0[c]	4.1[a]	4.1[a]
θ_0	K	767[a]	417[b]	1100[c]	905[a]	871[a]
γ_0		1.36[a]	1.41[b]	1.3[c]	1.57[a]	1.57[a]
n		2	2	3	5	5
q		1.7[a]	0.5[b]	1.0[c]	1.1[a]	1.1[a]
V_0	cm^3mol^{-1}	11.24[a]	12.256[b]	14.483[c]	24.45[a]	25.49[a]

[a] from ref. [114].

[b] from ref. [106].

[c] from ref. [109].

Table S5: **Relevant densities of all mineral components**. These densities are calculated at temperatures of 4000 K and pressures 130 GPa, and applied for the calculation of all density profiles (e.g., Figs. 2, 5). See Suppl. Text for details.

Component	Density at 130 GPa
$MgSiO_3$	5253.0 kg/m^3
$FeSiO_3$	6546.5 kg/m^3
MgO	5128.7 kg/m^3
FeO	8460.1 kg/m^3
SiO_2	5377.3 kg/m^3

REFERENCES AND NOTES

1. V. Solomatov, in *Treatise on Geophysics (Second Edition),* G. Schubert, Ed. (Elsevier, 2015), pp. 81–104.

2. S. Labrosse, J. W. Hernlund, N. Coltice, A crystallizing dense magma ocean at the base of the Earth's mantle. *Nature* **450**, 866–869 (2007).

3. R. Caracas, K. Hirose, R. Nomura, M. D. Ballmer, Melt–crystal density crossover in a deep magma ocean. *Earth Planet. Sci. Lett.* **516**, 202–211 (2019).

4. J. L. Mosenfelder, P. D. Asimow, D. J. Frost, D. C. Rubie, T. J. Ahrens, The $MgSiO_3$ system at high pressure: Thermodynamic properties of perovskite, postperovskite, and melt from global inversion of shock and static compression data. *J. Geophys. Res. Solid Earth* **114**, B01203 (2009).

5. W. F. McDonough, S.-S. Sun, The composition of the Earth. *Chem. Geol.* **120**, 223–253 (1995).

6. H. Deng, M. D. Ballmer, C. Reinhardt, M. M. M. Meier, L. Mayer, J. Stadel, F. Benitez, Primordial Earth mantle heterogeneity caused by the moon-forming giant impact? *Astrophys. J.* **887**, 211 (2019).

7. Q. Yuan, M. Li, S. J. Desch, B. Ko, H. Deng, E. J. Garnero, T. S. Gabriel, J. A. Kegerreis, Y. Miyazaki, V. Eke, P. D. Asimow, Moon-forming impactor as a source of Earth's basal mantle anomalies. *Nature* **623**, 95–99 (2023).

8. L. Xie, A. Yoneda, D. Yamazaki, G. Manthilake, Y. Higo, Y. Tange, N. Guignot, A. King, M. Scheel, D. Andrault, Formation of bridgmanite-enriched layer at the top lower-mantle during magma ocean solidification. *Nat. Commun.* **11**, 548 (2020).

9. M. D. Ballmer, D. L. Lourenço, K. Hirose, R. Caracas, R. Nomura, Reconciling magma-ocean crystallization models with the present-day structure of the Earth's mantle. *Geochem. Geophys. Geosyst.* **18**, 2785–2806 (2017).

10. C.-E. Boukaré, E. Parmentier, S. W. Parman, Timing of mantle overturn during magma ocean solidification. *Earth Planet. Sci. Lett.* **491**, 216–225 (2018).

11. L. B. Ziegler, D. R. Stegman, Implications of a long-lived basal magma ocean in generating Earth's ancient magnetic field. *Geochem. Geophys. Geosyst.* **14**, 4735–4742 (2013).

12. V. Lherm, M. Nakajima, E. G. Blackman, Thermal and magnetic evolution of an Earth-like planet with a basal magma ocean. *Phys. Earth Planet. Inter.* **356**, 107267 (2024).

13. H. Samuel, M. Drilleau, A. Rivoldini, Z. Xu, Q. Huang, R. F. Garcia, V. Lekić, J. C. Irving, J. Badro, P. H. Lognonné, J. A. D. Connolly, T. Kawamura, T. Gudkova, W. B. Banerdt, Geophysical evidence for an enriched molten silicate layer above Mars's core. *Nature* **622**, 712–717 (2023).

14. A. Khan, D. Huang, C. Durán, P. A. Sossi, D. Giardini, M. Murakami, Evidence for a liquid silicate layer atop the Martian core. *Nature* **622**, 718–723 (2023).

15. H. Samuel, M. D. Ballmer, S. Padovan, N. Tosi, A. Rivoldini, A. C. Plesa, The thermo-chemical evolution of Mars with a strongly stratified mantle. *J. Geophys. Res. Planets* **126**, e2020JE006613 (2021).

16. R. C. Weber, P.-Y. Lin, E. J. Garnero, Q. Williams, P. Lognonné, Seismic detection of the lunar core. *Science* **331**, 309–312 (2011).

17. G. Kraettli, M. W. Schmidt, C. Liebske, Fractional crystallization of a basal lunar magma ocean: A dense melt-bearing garnetite layer above the core? *Icarus* **371**, 114699 (2022).

18. S. Russell, J. C. E. Irving, L. Jagt, S. Cottaar, Evidence for a kilometer-scale seismically slow layer atop the core-mantle boundary from normal modes. *Geophys. Res. Lett.* **50**, e2023GL105684 (2023).

19. C. E. Boukaré, Y. Ricard, G. Fiquet, Thermodynamics of the MgO-FeO-SiO_2 system up to 140 GPa: Application to the crystallization of Earth's magma ocean. *J. Geophys. Res. Solid Earth* **120**, 6085–6101 (2015).

20. M. Le Bars, A. Davaille, Whole layer convection in a heterogeneous planetary mantle. *J. Geophys. Res.* **109**, B03403 (2004).

21. N. Tosi, A. C. Plesa, D. Breuer, Overturn and evolution of a crystallized magma ocean: A numerical parameter study for Mars. *J. Geophys. Res. Planets* **118**, 1512–1528 (2013).

22. F. Deschamps, P. J. Tackley, Searching for models of thermo-chemical convection that explain probabilistic tomography. II—Influence of physical and compositional parameters. *Phys. Earth Planet. Inter.* **176**, 1–18 (2009).

23. A. K. McNamara, S. Zhong, Thermochemical structures within a spherical mantle: Superplumes or piles? *J. Geophys. Res.* **109**, B07402 (2004).

24. T. Nakagawa, P. J. Tackley, F. Deschamps, J. A. D. Connolly, The influence of MORB and harzburgite composition on thermo-chemical mantle convection in a 3-D spherical shell with self-consistently calculated mineral physics. *Earth Planet. Sci. Lett.* **296**, 403–412 (2010).

25. J. K. Wicks, J. M. Jackson, W. Sturhahn, D. Zhang, Sound velocity and density of magnesiowüstites: Implications for ultralow-velocity zone topography. *Geophys. Res. Lett.* **44**, 2148–2158 (2017).

26. S. Yu, E. J. Garnero, Ultralow velocity zone locations: A global assessment. *Geochem. Geophys. Geosyst.* **19**, 396–414 (2018).

27. J. Trampert, F. Deschamps, J. S. Resovsky, D. Yuen, Probabilistic tomography maps chemical heterogeneities throughout the lower mantle. *Science* **306**, 853–856 (2004).

28. K. Vilella, T. Bodin, C.-E. Boukaré, F. Deschamps, J. Badro, M. D. Ballmer, Y. Li, Constraints on the composition and temperature of LLSVPs from seismic properties of lower mantle minerals. *Earth Planet. Sci. Lett.* **554**, 116685 (2021).

29. F. D. Richards, M. J. Hoggard, S. Ghelichkhan, P. Koelemeijer, H. C. Lau, Geodynamic, geodetic, and seismic constraints favour deflated and dense-cored LLVPs. *Earth Planet. Sci. Lett.* **602**, 117964 (2023).

30. H. C. P. Lau, J. X. Mitrovica, J. L. Davis, J. Tromp, H.-Y. Yang, D. Al-Attar, Tidal tomography constrains Earth's deep-mantle buoyancy. *Nature* **551**, 321–326 (2017).

31. S. Cottaar, V. Lekic, Morphology of seismically slow lower-mantle structures. *Geophys. J. Int.* **207**, 1122–1136 (2016).

32. Y. Miyazaki, J. Korenaga, On the timescale of magma ocean solidification and its chemical consequences: 2. Compositional differentiation under crystal accumulation and matrix compaction. *J. Geophys. Res. Solid Earth* **124**, 3399–3419 (2019).

33. F. Nabiei, J. Badro, C. É. Boukaré, C. Hébert, M. Cantoni, S. Borensztajn, N. Wehr, P. Gillet, Investigating magma ocean solidification on Earth through laser-heated diamond anvil cell experiments. *Geophys. Res. Lett.* **48**, e2021GL092446 (2021).

34. T. Lay, J. Hernlund, B. A. Buffett, Core-mantle boundary heat flow. *Nat. Geosci.* **1**, 25–32 (2008).

35. R. Nomura, K. Hirose, K. Uesugi, Y. Ohishi, A. Tsuchiyama, A. Miyake, Y. Ueno, Low core-mantle boundary temperature inferred from the solidus of pyrolite. *Science* **343**, 522–525 (2014).

36. D. Alfè, M. J. Gillan, G. D. Price, Composition and temperature of the Earth's core constrained by combining ab initio calculations and seismic data. *Earth Planetary Sci. Lett.* **195**, 91–98 (2002).

37. N. Coltice, M. Moreira, J. Hernlund, S. Labrosse, Crystallization of a basal magma ocean recorded by helium and neon. *Earth Planet. Sci. Lett.* **308**, 193–199 (2011).

38. D. J. Frost, C. Liebske, F. Langenhorst, C. A. McCammon, R. G. Trønnes, D. C. Rubie, Experimental evidence for the existence of iron-rich metal in the Earth's lower mantle. *Nature* **428**, 409–412 (2004).

39. R. Huang, T. Boffa Ballaran, C. A. McCammon, N. Miyajima, D. Dolejš, D. J. Frost, The composition and redox state of bridgmanite in the lower mantle as a function of oxygen fugacity. *Geochim. Cosmochim. Acta* **303**, 110–136 (2021).

40. J. P. Brandenburg, P. E. van Keken, Deep storage of oceanic crust in a vigorously convecting mantle. *J. Geophys. Res. Solid Earth* **112**, B06403 (2007).

41. J. Yan, M. D. Ballmer, P. J. Tackley, The evolution and distribution of recycled oceanic crust in the Earth's mantle: Insight from geodynamic models. *Earth Planet. Sci. Lett.* **537**, 116171 (2020).

42. D. L. Lourenço, A. B. Rozel, M. D. Ballmer, P. J. Tackley, Plutonic-squishy lid: A new global tectonic regime generated by intrusive magmatism on Earth-like planets. *Geochem. Geophys. Geosyst.* **21**, e2019GC008756 (2020).

43. T. E. Johnson, M. Brown, B. J. P. Kaus, J. A. VanTongeren, Delamination and recycling of Archaean crust caused by gravitational instabilities. *Nat. Geosci.* **7**, 47–52 (2014).

44. W. B. Moore, A. A. G. Webb, Heat-pipe earth. *Nature* **501**, 501–505 (2013).

45. E. Sizova, T. Gerya, M. Brown, L. L. Perchuk, Subduction styles in the Precambrian: Insight from numerical experiments. *Lithos* **116**, 209–229 (2010).

46. J. van Hunen, J.-F. Moyen, Archean subduction: Fact or fiction? *Annu. Rev. Earth Planet. Sci.* **40**, 195–219 (2012).

47. R. A. Cabral, M. G. Jackson, E. F. Rose-Koga, K. T. Koga, M. J. Whitehouse, M. A. Antonelli, J. Farquhar, J. M. D. Day, E. H. Hauri, Anomalous sulphur isotopes in plume lavas reveal deep mantle storage of Archaean crust. *Nature* **496**, 490–493 (2013).

48. T. M. Kusky, J.-H. Li, R. D. Tucker, The Archean Dongwanzi ophiolite complex, North China Craton: 2.505-billion-year-old oceanic crust and mantle. *Science* **292**, 1142–1145 (2001).

49. J. Korenaga, Crustal evolution and mantle dynamics through Earth history. *Philos. Trans. A Math. Phys. Eng. Sci.* **376**, 20170408 (2018).

50. J. C. Rosas, J. Korenaga, Archaean seafloors shallowed with age due to radiogenic heating in the mantle. *Nat. Geosci.* **14**, 51–56 (2021).

51. C. Herzberg, K. Condie, J. Korenaga, Thermal history of the Earth and its petrological expression. *Earth Planet. Sci. Lett.* **292**, 79–88 (2010).

52. D. Andrault, G. Pesce, M. A. Bouhifd, N. Bolfan-Casanova, J.-M. Hénot, M. Mezouar, Melting of subducted basalt at the core-mantle boundary. *Science* **344**, 892–895 (2014).

53. L. Bindi, S.-H. Shim, T. G. Sharp, X. Xie, Evidence for the charge disproportionation of iron in extraterrestrial bridgmanite. *Sci. Adv.* **6**, eaay7893 (2020).

54. M. D. Ballmer, C. Houser, J. W. Hernlund, R. M. Wentzcovitch, K. Hirose, Persistence of strong silica-enriched domains in the Earth's lower mantle. *Nat. Geosci.* **10**, 236–240 (2017).

55. Z. Wu, J. Song, G. Zhao, Z. Pan, Water-induced mantle overturns leading to the origins of Archean continents and subcontinental lithospheric mantle. *Geophys. Res. Lett.* **50**, e2023GL105178 (2023).

56. F. Gaillard, B. Scaillet, N. T. Arndt, Atmospheric oxygenation caused by a change in volcanic degassing pressure. *Nature* **478**, 229–232 (2011).

57. T. W. Lyons, C. T. Reinhard, N. J. Planavsky, The rise of oxygen in Earth's early ocean and atmosphere. *Nature* **506**, 307–315 (2014).

58. M. M. Hirschmann, The deep Earth oxygen cycle: Mass balance considerations on the origin and evolution of mantle and surface oxidative reservoirs. *Earth Planet. Sci. Lett.* **619**, 118311 (2023).

59. E. A. Sperling, G. P. Halverson, A. H. Knoll, F. A. Macdonald, D. T. Johnston, A basin redox transect at the dawn of animal life. *Earth Planet. Sci. Lett.* **371-372**, 143–155 (2013).

60. T. W. Becker, J. B. Kellogg, R. J. O'Connell, Thermal constraints on the survival of primitive blobs in the lower mantle. *Earth Planet. Sci. Lett.* **171**, 351–365 (1999).

61. A. J. P. Gülcher, M. D. Ballmer, P. J. Tackley, Coupled dynamics and evolution of primordial and recycled heterogeneity in Earth's lower mantle. *Solid Earth* **12**, 2087–2107 (2021).

62. N. Tsujino, D. Yamazaki, Y. Nishihara, T. Yoshino, Y. Higo, Y. Tange, Viscosity of bridgmanite determined by in situ stress and strain measurements in uniaxial deformation experiments. *Sci. Adv.* **8**, eabm1821 (2022).

63. H. Fei, M. D. Ballmer, U. Faul, N. Walte, W. Cao, T. Katsura, Variation in bridgmanite grain size accounts for the mid-mantle viscosity jump. *Nature* **620**, 794–799 (2023).

64. L. Waszek, N. C. Schmerr, M. D. Ballmer, Global observations of reflectors in the mid-mantle with implications for mantle structure and dynamics. *Nat. Commun.* **9**, 385 (2018).

65. Y. Fukao, M. Obayashi, Subducted slabs stagnant above, penetrating through, and trapped below the 660 km discontinuity. *J. Geophys. Res. Solid Earth* **118**, 5920–5938 (2013).

66. S. W. French, B. Romanowicz, Broad plumes rooted at the base of the Earth's mantle beneath major hotspots. *Nature* **525**, 95–99 (2015).

67. R. Nomura, K. Hirose, N. Sata, Y. Ohishi, Precise determination of post-stishovite phase transition boundary and implications for seismic heterogeneities in the mid-lower mantle. *Phys. Earth Planet. Inter.* **183**, 104–109 (2010).

68. E. Mulyukova, B. Steinberger, M. Dabrowski, S. V. Sobolev, Survival of LLSVPs for billions of years in a vigorously convecting mantle: Replenishment and destruction of chemical anomaly. *J. Geophys. Res. Solid Earth* **120**, 3824–3847 (2015).

69. A. L. Bull, A. K. McNamara, J. Ritsema, Synthetic tomography of plume clusters and thermochemical piles. *Earth Planet. Sci. Lett.* **278**, 152–162 (2009).

70. J. W. Hernlund, C. Houser, On the statistical distribution of seismic velocities in Earth's deep mantle. *Earth Planet. Sci. Lett.* **265**, 423–437 (2008).

71. S. Cottaar, V. Lekic, Morphology of seismically slow lower-mantle structures. *Geophys. J. Int.* **207**, 1122–1136 (2016).

72. A. Davaille, B. Romanowicz, Deflating the LLSVPs: Bundles of mantle thermochemical plumes rather than thick stagnant "piles". *Tectonics* **39**, e2020TC006265 (2020).

73. S. Pachhai, M. Li, M. S. Thorne, J. Dettmer, H. Tkalčić, Internal structure of ultralow-velocity zones consistent with origin from a basal magma ocean. *Nat. Geosci.* **15**, 79–84 (2022).

74. J. M. R. Muir, J. P. Brodholt, Elastic properties of ferrous bearing $MgSiO_3$ and their relevance to ULVZs. *Geophys. J. Int.* **201**, 496–504 (2015).

75. I. S. Puchtel, J. Blichert-Toft, M. Touboul, M. F. Horan, R. J. Walker, The coupled ^{182}W-^{142}Nd record of early terrestrial mantle differentiation. *Geochem. Geophys. Geosyst.* **17**, 2168–2193 (2016).

76. J. R. Reimink, A. Mundl-Petermeier, R. W. Carlson, S. B. Shirey, R. J. Walker, D. G. Pearson, Tungsten isotope composition of Archean crustal reservoirs and implications for terrestrial $\mu^{182}W$ evolution. *Geochem. Geophys. Geosyst.* **21**, e2020GC009155 (2020).

77. E. Hyung, S. B. Jacobsen, The $^{142}Nd/^{144}Nd$ variations in mantle-derived rocks provide constraints on the stirring rate of the mantle from the Hadean to the present. *Proc. Natl. Acad. Sci. U.S.A.* **117**, 14738–14744 (2020).

78. A. Mundl-Petermeier, R. Walker, R. Fischer, V. Lekic, M. Jackson, M. Kurz, Anomalous ^{182}W in high $^{3}He/^{4}He$ ocean island basalts: Fingerprints of Earth's core? *Geochim. Cosmochim. Acta* **271**, 194–211 (2020).

79. M. Jackson, J. Konter, T. Becker, Primordial helium entrained by the hottest mantle plumes. *Nature* **542**, 340–343 (2017).

80. A. W. Hofmann, Mantle geochemistry: The message from oceanic volcanism. *Nature* **385**, 219–229 (1997).

81. K. Hirose, G. Morard, R. Sinmyo, K. Umemoto, J. Hernlund, G. Helffrich, S. Labrosse, Crystallization of silicon dioxide and compositional evolution of the Earth's core. *Nature* **543**, 99–102 (2017).

82. S. Labrosse, J. W. Hernlund, K. Hirose, "Fractional melting and freezing in the deep mantle and implications for the formation of a basal magma ocean" in *The Early Earth: Accretion and Differentiation* (AGU Publications, 2015), pp. 123–142.

83. T. Rolf, M. Weller, A. Gülcher, P. Byrne, J. G. O'Rourke, R. Herrick, E. Bjonnes, A. Davaille, R. Ghail, C. Gillmann, A. C. Plesa, S. Smrekar, Dynamics and evolution of Venus' mantle through time. *Space Sci. Rev.* **218**, 70 (2022).

84. J. G. O'Rourke, Venus: A thick basal magma ocean may exist today. *Geophys. Res. Lett.* **47**, e2019GL086126 (2020).

85. A.-C. Plesa, M. Wieczorek, M. Knapmeyer, A. Rivoldini, M. Walterova, D. Breuer, "Interior dynamics and thermal evolution of Mars – a geodynamic perspective" in *Advances in Geophysics*. (Elsevier, 2022), vol. 63, pp. 179–230.

86. M. Laneuville, M. Wieczorek, D. Breuer, J. Aubert, G. Morard, T. Rückriemen, A long-lived lunar dynamo powered by core crystallization. *Earth Planet. Sci. Lett.* **401**, 251–260 (2014).

87. A. Boujibar, N. Bolfan-Casanova, D. Andrault, M. Ali Bouhifd, N. Trcera, Incorporation of Fe^{2+} and Fe^{3+} in bridgmanite during magma ocean crystallization. *Am. Mineral.* **101**, 1560–1570 (2016).

88. I. Jackson, S. M. Rigden, Analysis of PVT data: Constraints on the thermoelastic properties of high-pressure minerals. *Phys. Earth Planet. Inter.* **96**, 85–112 (1996).

89. K. J. Matthews, K. T. Maloney, S. Zahirovic, S. E. Williams, M. Seton, R. D. Müller, Global plate boundary evolution and kinematics since the late Paleozoic. *Global Planet. Change* **146**, 226–250 (2016).

90. M. W. Hounslow, M. Domeier, A. J. Biggin, Subduction flux modulates the geomagnetic polarity reversal rate. *Tectonophysics* **742-743**, 34–49 (2018).

91. T. Nakagawa, P. J. Tackley, Influence of combined primordial layering and recycled MORB on the coupled thermal evolution of Earth's mantle and core. *Geochem. Geophys. Geosyst.* **15**, 619–633 (2014).

92. R. Boehler, Melting temperature of the Earth's mantle and core: Earth's thermal structure. *Annu. Rev. Earth Planet. Sci.* **24**, 15–40 (1996).

93. H. Piet, J. Badro, F. Nabiei, T. Dennenwaldt, S.-H. Shim, M. Cantoni, C. Hébert, P. Gillet, Spin and valence dependence of iron partitioning in Earth's deep mantle. *Proc. Natl. Acad. Sci. U.S.A.* **113**, 11127–11130 (2016).

94. R. Sinmyo, K. Hirose, Iron partitioning in pyrolitic lower mantle. *Phys. Chem. Miner.* **40**, 107–113 (2013).

95. S. Tateno, K. Hirose, Y. Ohishi, Melting experiments on peridotite to lowermost mantle conditions. *J. Geophys. Res. Solid Earth* **119**, 4684–4694 (2014).

96. D. Andrault, S. Petitgirard, G. L. Nigro, J.-L. Devidal, G. Veronesi, G. Garbarino, M. Mezouar, Solid-liquid iron partitioning in Earth's deep mantle. *Nature* **487**, 354–357 (2012).

97. S. Fu, J. Yang, Y. Zhang, J. Liu, E. Greenberg, V. B. Prakapenka, T. Okuchi, J.-F. Lin, Melting behavior of the lower-mantle ferropericlase across the spin crossover: Implication for the ultra-low velocity zones at the lowermost mantle. *Earth Planet. Sci. Lett.* **503**, 1–9 (2018).

98. M. Murakami, K. Hirose, N. Sata, Y. Ohishi, Post-perovskite phase transition and mineral chemistry in the pyrolitic lowermost mantle. *Geophys. Res. Lett.* **32**, doi:10.1029/2004GL021956 (2005).

99. M. Desiderio, M. D. Ballmer, Ancient stratified thermochemical piles due to high intrinsic viscosity. *Geophys. Res. Lett.* **51**, e2024GL110006 (2024).

100. R. I. Citron, D. L. Lourenço, A. J. Wilson, A. G. Grima, S. A. Wipperfurth, M. L. Rudolph, S. Cottaar, L. G. Montési, Effects of heat-producing elements on the stability of deep mantle thermochemical piles. *Geochem. Geophys. Geosyst.* **21**, e2019GC008895 (2020).

101. E. Tan, M. Gurnis, Compressible thermochemical convection and application to lower mantle structures. *J. Geophys. Res. Solid Earth* **112**, B06304 (2007).

102. N. Tosi, D. A. Yuen, N. de Koker, R. M. Wentzcovitch, Mantle dynamics with pressure- and temperature-dependent thermal expansivity and conductivity. *Phys. Earth Planet. Inter.* **217**, 48–58 (2013).

103. Y. Fei, L. Zhang, A. Corgne, H. Watson, A. Ricolleau, Y. Meng, V. Prakapenka, Spin transition and equations of state of (Mg, Fe)O solid solutions. *Geophys. Res. Lett.* **34**, (2007).

104. M. Matsui, E. Ito, D. Yamazaki, T. Yoshino, X. Guo, S. Shan, Y. Higo, K.-I. Funakoshi, Static compression of $(Mg_{0.83}, Fe_{0.17})O$ and $(Mg_{0.75}, Fe_{0.25})O$ ferropericlase up to 58 GPa at 300, 700, and 1100 K. *Am. Mineral.* **97**, 176–183 (2012).

105. T. Komabayashi, K. Hirose, Y. Nagaya, E. Sugimura, Y. Ohishi, High-temperature compression of ferropericlase and the effect of temperature on iron spin transition. *Earth Planet. Sci. Lett.* **297**, 691–699 (2010).

106. R. A. Fischer, A. J. Campbell, G. A. Shofner, O. T. Lord, P. Dera, V. B. Prakapenka, Equation of state and phase diagram of FeO. *Earth Planet. Sci. Lett.* **304**, 496–502 (2011).

107. S. Lundin, K. Catalli, J. Santillan, S.-H. Shim, V. Prakapenka, M. Kunz, Y. Meng, Effect of Fe on the equation of state of mantle silicate perovskite over 1 Mbar. *Phys. Earth Planet. Inter.* **168**, 97–102 (2008).

108. S. M. Dorfman, Y. Meng, V. B. Prakapenka, T. S. Duffy, Effects of Fe-enrichment on the equation of state and stability of $(Mg, Fe)SiO_3$ perovskite. *Earth Planet. Sci. Lett.* **361**, 249–257 (2013).

109. N. Sun, W. Shi, Z. Mao, C. Zhou, V. B. Prakapenka, High pressure-temperature study on the thermal equations of state of seifertite and$CaCl_2$-type SiO_2. *J. Geophys. Res. Solid Earth* **124**, 12620–12630 (2019).

110. J. Buchen, H. Marquardt, K. Schulze, S. Speziale, T. Boffa Ballaran, N. Nishiyama, M. Hanfland, Equation of state of polycrystalline stishovite across the tetragonal-orthorhombic phase transition. *J. Geophys. Res. Solid Earth* **123**, 7347–7360 (2018).

111. R. A. Fischer, A. J. Campbell, B. A. Chidester, D. M. Reaman, E. C. Thompson, J. S. Pigott, V. B. Prakapenka, J. S. Smith, Equations of state and phase boundary for stishovite and $CaCl_2$-type SiO_2. *Am. Mineral.* **103**, 792–802 (2018).

112. D. G. van der Meer, W. Spakman, D. J. J. van Hinsbergen, M. L. Amaru, T. H. Torsvik, Towards absolute plate motions constrained by lower-mantle slab remnants. *Nat. Geosci.* **3**, 36–40 (2010).

113. M. Domeier, P. V. Doubrovine, T. H. Torsvik, W. Spakman, A. L. Bull, Global correlation of lower mantle structure and past subduction. *Geophys. Res. Lett.* **43**, 4945–4953 (2016).

114. L. Stixrude, C. Lithgow-Bertelloni, Thermodynamics of mantle minerals—II. Phase equilibria. *Geophys. J. Int.* **184**, 1180–1213 (2011).
